# Investigating the effects of Ginkgo biloba leaf extract on cognitive function in Alzheimer's disease

**DOI:** 10.1111/cns.14914

**Published:** 2024-09-05

**Authors:** Cheng Zhu, Jie Liu, Jixin Lin, Jiaxi Xu, Enyan Yu

**Affiliations:** ^1^ School of Mental Health, Zhejiang Provincial Clinical Research Center for Mental Disorders, The Affiliated Wenzhou Kangning Hospital Wenzhou Medical University Wenzhou China; ^2^ School of Mental Health Wenzhou Medical University Wenzhou China; ^3^ The Second People's Hospital of Chuzhou Sleep Disorders Department Chuzhou China; ^4^ Second Clinical Medicine Faculty Zhejiang Chinese Medical University Hangzhou China; ^5^ General Psychiatric Department Tongde Hospital of Zhejiang Province Hangzhou China; ^6^ Clinical Psychology Department Zhejiang Cancer Hospital Hangzhou China

**Keywords:** Alzheimer's disease, Ginkgo biloba leaf extract, kaempferol, luteolin, PI3K/AKT/NF‐κB signaling pathway, quercetin

## Abstract

**Aims:**

Alzheimer's disease (AD) is a neurodegenerative disorder with limited treatment options. This study aimed to investigate the therapeutic effects of Ginkgo biloba leaf extract (GBE) on AD and explore its potential mechanisms of action.

**Methods:**

Key chemical components of GBE, including quercetin, luteolin, and kaempferol, were identified using network pharmacology methods. Bioinformatics analysis revealed their potential roles in AD through modulation of the PI3K/AKT/NF‐κB signaling pathway.

**Results:**

Mouse experiments demonstrated that GBE improved cognitive function, enhanced neuronal morphology, and reduced serum inflammatory factors. Additionally, GBE modulated the expression of relevant proteins and mRNA.

**Conclusion:**

GBE shows promise as a potential treatment for AD. Its beneficial effects on cognitive function, neuronal morphology, and inflammation may be attributed to its modulation of the PI3K/AKT/NF‐κB signaling pathway. These findings provide experimental evidence for the application of Ginkgo biloba leaf in AD treatment and highlight its potential mechanisms of action.

## Graphical abstract

The Ginkgo biloba leaf extract (GBE) study demonstrates its promise as an Alzheimer's treatment, significantly improving cognitive function and neuronal morphology. The effects are mediated through modulation of the PI3K/AKT/NF‐κB signaling pathway, which impacts inflammation and neuronal health, offering a potential new avenue for therapeutic intervention in neurodegenerative disorders.

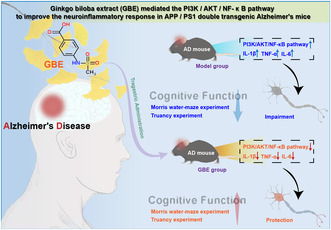

## 1 INTRODUCTION

Alzheimer's disease (AD) is a neurodegenerative disorder characterized by memory loss and cognitive decline, affecting millions of elderly people worldwide.^1^, ^2^, ^3^ With the global population aging, the number of AD patients is expected to continue increasing in the coming decades.^4^, ^5^ Current hypotheses on the pathogenesis of AD include the deposition of amyloid proteins and neurofibrillary tangles, abnormal levels of oxidative stress, and insulin resistance.^6^, ^7^, ^8^ The pathological features of AD include the deposition of amyloid‐like proteins and the formation of neurofibrillary tangles in the brain, which result in impaired neuronal function and death.^9^, ^10^, ^11^

Currently, the treatment of AD primarily focuses on symptom relief rather than the prevention or reversal of disease progression.^6^, ^12^, ^13^ Although several drugs have been approved for the treatment of AD, their effectiveness is limited and often accompanied by adverse reactions.^14^, ^15^, ^16^ Numerous studies focusing on amyloid‐related targeted drugs have indicated that the clearance of amyloid protein does not lead to a significant improvement in cognitive function, as evidenced by research findings.^17^, ^18^ Therefore, the search for more effective and safe treatment methods is the current focus of AD research.^19^, ^20^, ^21^

Current drugs used to treat AD, such as acetylcholinesterase inhibitors and NMDA receptor antagonists, can only partially relieve symptoms and cannot stop or reverse the progression of the disease.^22^, ^23^, ^24^ Moreover, the side effects of these drugs, including nausea, dizziness, and cardiac issues, limit their use.^25^, ^26^ Therefore, researchers have started to focus on traditional herbs and natural extracts, such as Ginkgo biloba extract (GBE), as potential alternative therapies for AD.^27^ GBE has long been used in traditional medicine to improve brain circulation and function.^28^ GBE contains various active compounds, such as flavonoids and terpenoids, which have shown potential for neuroprotection.^29^

In recent years, GBE has shown potential in improving AD‐related symptoms.^30^ Several studies have demonstrated that GBE can improve cognitive function, reduce amyloid deposition, and inhibit the formation of neurofibrillary tangles.^30^, ^31^ The active components in GBE, such as flavonoids and terpenoids, are considered key to its neuroprotective effects.^32^, ^33^ These compounds have been shown to act through various mechanisms, including antioxidant, anti‐inflammatory, and modulation of neurotransmitters.^34^, ^35^, ^36^ Additionally, GBE has also been found to improve brain circulation, further promoting brain health.

Network pharmacology is an emerging research approach that explores the mechanism of action and potential therapeutic effects of drugs by integrating complex interactions between drugs, targets, and diseases.^37^, ^38^, ^39^ In AD research, network pharmacology methods have been used to identify potentially beneficial chemical components and their target interactions in GBE for AD.^38^, ^40^, ^41^ By combining bioinformatics tools and experimental data, researchers can construct network models of components‐targets‐diseases to reveal the potential mechanisms of GBE in AD treatment.^42^, ^43^, ^44^ This approach not only helps to understand the complex actions of GBE but also provides a valuable research tool for the development of new treatment strategies.^45^, ^46^, ^47^

The main objective of this study is to explore the potential role and mechanism of GBE in the treatment of AD. We plan to use network pharmacology methods to screen candidate genes and chemical components in GBE that may alleviate AD symptoms and evaluate their effects on cognitive function and neuronal morphology using a mouse model. Furthermore, this study will investigate how GBE exerts its effects by regulating the PI3K/AKT/NF‐κB signaling pathway and influencing the release of inflammatory factors. The results of this study are expected to provide new strategies for the treatment of AD and enhance our understanding of the pathological mechanisms of AD. By deeply studying the mechanism of GBE, this research will provide important scientific evidence for the development of more effective and safer AD treatments. Clinically, this could not only improve the quality of life for AD patients but also lead to breakthroughs in the field.

## 2 MATERIALS AND METHODS

### 2.1 Identification and screening of active chemical components and target proteins of Ginkgo biloba leaf

In this study, a comprehensive screening and analysis of the active chemical components and target proteins in Ginkgo biloba leaf were conducted using the TCMSP database (https://old.tcmsp‐e.com/tcmsp.php). Initially, relevant information on Ginkgo biloba leaf was retrieved from the TCMSP database, with criteria set at an oral bioavailability (OB) >30% and drug‐likeness (DL) >0.18, in order to identify key active chemical components in Ginkgo biloba leaf. This screening process aimed to identify components with high biological availability and pharmaceutical potential, laying a solid foundation for subsequent research. Among the screened chemical components, those without clear target proteins or with duplicate records in the database were further excluded to ensure the accuracy and reliability of the research results. Subsequently, a detailed analysis of the remaining active components' target proteins was performed using the TCMSP database for retrieval while eliminating all duplicates. The purpose of this step was to clarify the potential biological mechanisms and target proteins of these chemical components, providing a basis for further pharmacological and pharmaceutical studies.

### 2.2 Bioinformatics screening of Gingko leaf‐regulated Alzheimer's disease differential genes

To identify potential regulatory targets of Gingko leaf in AD, we first obtained an AD‐related expression profiling dataset (GSE37263) from the Gene Expression Omnibus (GEO) database (https://www.ncbi.nlm.nih.gov/gds). This dataset included 8 control samples and 8 AD samples. Differential gene analysis was performed on these samples using the “limma” package in the R language, with the screening criteria set as |log_2_(Fold Change)| > 1 and *p* < 0.05. Subsequently, we searched for target genes related to AD in the OMIM database (https://omim.org/) and the GeneCards database (https://www.genecards.org/), with a relevance score >20 as the filtering condition. The differential genes selected from the GSE37263 dataset were merged with these target genes to determine disease targets for AD. Furthermore, we conducted an intersection analysis between these disease targets and the target genes of active components in Gingko leaf to identify potential targets regulated by Gingko leaf in AD. Finally, for the visual presentation of these potential targets, we performed a Venn analysis using the online analysis website (https://www.xiantao.love/) and visualized the results accordingly.

### 2.3 Construction of the “Ginkgo biloba leaf‐compound‐target‐Alzheimer's disease” network

To investigate the potential impact of Ginkgo biloba leaf components on AD, this study utilized Cytoscape 3.7.2 software to construct a “Drug‐Compound‐Target‐Disease” network model. The purpose of this network model is to visually display the main active components of Ginkgo biloba leaf and their potential target interactions, as well as their association with AD. During the network construction process, particular attention was paid to the connectivity of nodes, as it serves as a crucial indicator of the importance of nodes within the network. To facilitate this, the cytoNCA plugin in Cytoscape was employed, using the Betweenness Centrality (BC), Closeness Centrality (CC), and c algorithms to calculate the Degree value for each node. These calculations aid in identifying key nodes in the network, which are likely to play a central role in the regulation of AD by Ginkgo biloba leaf.

### 2.4 Node sorting in protein–protein interaction networks

A protein–protein interaction (PPI) analysis was conducted to identify candidate target proteins in Ginkgo biloba leaves. The analysis was performed using the STRING database (https://string‐db.org), with the species restriction set to “Homo sapiens”. The basic settings included the highest confidence level (0.900) and the removal of free nodes, while the remaining parameters were set to their default values. The resulting PPI network, representing the interaction between drugs and diseases, was imported into Cytoscape 3.7.2. The cytoNCA plugin, incorporating the BC, CC, and DC algorithms, was utilized to compute the Degree values and determine the key target proteins. Subsequently, the nodes in the PPI network were sorted based on their Degree values to obtain the crucial genes.

### 2.5 Functional enrichment analysis of Ginkgo biloba leaf‐regulated Alzheimer's disease targets

In this study, to gain a deeper understanding of the biological functions and mechanisms of Ginkgo biloba leaf‐regulated AD candidate targets, we employed functional enrichment analysis. Specifically, we utilized the online analysis platform Xiantao Academic (https://www.xiantao.love/) and the “ClusterProfiler” package in R (available at the Bioconductor website: http://www.bioconductor.org/packages/release/bioc/html/clusterProfiler.html) to perform comprehensive gene ontology (GO) and Kyoto Encyclopedia of Genes and Genomes (KEGG) enrichment analyses on the selected candidate targets. In GO analysis, we focused on three aspects: Biological Process (BP), Molecular Function (MF), and Cellular Component (CC). Through these analyses, we aimed to uncover the major roles of the candidate targets in cellular functions and signaling pathways, providing deeper insights into the potential therapeutic effects of Ginkgo biloba leaf in AD. All analyses were considered significantly enriched if the *p*‐value was <0.05. By integrating these comprehensive functional enrichment analyses, we aim to gain a more comprehensive understanding of the impact of active components in Ginkgo biloba leaf on AD, particularly at the molecular level. This will provide scientific evidence for future drug development and treatment strategies.

### 2.6 The APP/PS1 transgenic mouse model

This study used 6‐month‐old APP/PS1 transgenic mice as an AD model, with age‐matched C57BL/6 mice as the normal control group. All mice were provided by Beijing Vitonlihua Experimental Animal Co., Ltd., holding the experimental animal production license number [SCXK (Jing) 2019‐0011]. The experimental animals were housed in our university's Animal Center under the license number [SYXK (Zhe) 2019‐0022]. The experimental procedures strictly adhered to the “Guidelines for the Management and Use of Laboratory Animals” and were approved by the School's Animal Research Ethics Committee (Approval No: 20210322‐04).

All mice were housed in a barrier environment animal laboratory, with a room temperature maintained at (23 ± 2)°C and a relative humidity of (60 ± 2)%. The light–dark cycle was maintained at 12 h. The mice were fed with pellet‐like standard feed and had ad libitum access to water and food. The experimental mice were divided into 5 groups, each consisting of 10 mice, as follows: Control group (normal mice, administered with intragastric saline), Model group (APP/PS1 mice, administered with intragastric saline), GBE low‐dose group (APP/PS1 mice, administered with intragastric GBE dose of 1.5 mL/kg), GBE medium‐dose group (APP/PS1 mice, administered with intragastric GBE dose of 3 mL/kg), and GBE high‐dose group (APP/PS1 mice, administered with intragastric GBE dose of 6 mL/kg). All mice received daily intragastric administration once a day for a continuous period of 4 weeks.

### 2.7 Morris water maze test

The Morris water maze test is an experimental method used to assess spatial cognitive abilities in mice. It consists of a circular water tank with a diameter of 150 cm and a depth of 50 cm, maintained at a constant temperature of 25 ± 1°C. A cylindrical platform with a diameter of 9 cm and a height of 30 cm is placed in the third quadrant of the water tank. Before the experiment starts, the water level is adjusted to be 1 cm above the platform surface, and ink is added to make the water opaque, preventing direct visual recognition of the platform by the mice.

The experiment is divided into two stages: the acquisition phase and the probe trial. In the acquisition phase, the mice are placed into the water from different quadrants (one, two, and four) with their heads facing the wall. Each quadrant is tested once, and the mice undergo three training trials per day. If a mouse finds the platform within 60 s, the time from entering the water to climbing onto the platform, known as the escape latency, is recorded. If the mouse fails to find the platform within 60 s, it is guided to the platform and allowed to stay there for 10 s, with the escape latency recorded as 60 s. This training phase lasts for four days.

The probe trial, conducted on the day after the acquisition phase, involves the removal of the platform. The mice are placed into the water from the first quadrant, facing the wall, and the number of times they cross the original platform location within 60 s is recorded. A camera system placed above the water tank captures the swimming path of the mice, and data analysis and processing are performed using the SMART 3.0 spontaneous activity video analysis system. To ensure the accuracy of the experiment results, the surrounding environment, light conditions, and experimental procedures are kept consistent with those during the initial acquisition phase, minimizing the interference of external environmental factors.

### 2.8 Open field experiment

The open‐field experiment is a method used to assess spontaneous activity and exploratory ability in mice, primarily relying on the SMART 3.0 system for video analysis of spontaneous activity. This experiment takes place in a dimly lit observation box to simulate exploratory behavior in a natural environment. The open field analysis box used in the experiment has dimensions of 40 × 40 × 40 cm, and the video analysis software virtually divides it into a central grid and a peripheral area. At the beginning of the experiment, the mice are placed in the central grid, and their activity in the open field is observed and recorded for 5 min. Key observation measures during the experiment include the amount of time the mice spend in the central grid and the number of grid crossings, which reflect their level of spontaneous activity and exploratory behavior. After the experiment, the open field box is wiped clean to remove residual odors and prevent cross‐contamination between different mice, ensuring the integrity of subsequent animal tests.

### 2.9 ELISA

The ELISA experiment is a common method for determining the levels of inflammatory factors in serum. Blood samples from each group of mice were centrifuged at 3000 *g*/min for 10 min at 4°C in a precooled centrifuge. Following centrifugation, the clear serum from the top layer was aspirated using a micropipette, transferred to properly labeled new centrifuge tubes, and stored at −80°C for future use. Additionally, hippocampal tissues from the mouse groups were homogenized in prechilled PBS solution, then centrifuged at 5000 *g*/min for 10 min at 4°C to collect the supernatant, which was also stored at −80°C. During the ELISA experiment procedures, OD values were measured within 15 min at a wavelength of 450 nm as per the kit instructions.

### 2.10 Western blot

The hippocampal tissues from each group were sonicated and then mixed with RIPA and PMSF, followed by ice‐cold lysis for 30 min and centrifugation at 4°C for 15 min to collect the supernatant, which was stored at −80°C. The protein concentrations in each group were quantified using the BCA protein assay. Subsequently, immunoprecipitation and incubation with primary and secondary antibodies were carried out using standard procedures. Finally, ECL detection and analysis were performed using the Image Lab software. During membrane blocking, 5% milk was incubated for 2 h at room temperature on a shaker, followed by TBST washing. The primary antibodies used in the incubation included p‐PI3K (#17366), PI3K (#4257), p‐AKT (#4060), AKT (#9272), NF‐kB (#8242), p‐NF‐kB (#3033) antibodies provided by CST Biological Reagents Co., Ltd. with a dilution ratio of 1:1000 except for β‐actin (1:5000), and incubated overnight at 4°C on a shaker.

### 2.11 Golgi staining technique

Mouse brain tissues were washed three times in 0.1 M PBS. Subsequently, the slices were immersed in Golgi‐Cox solution (detailed below) and kept in a light‐avoiding environment at room temperature for 15–45 days. Afterward, the tissues were rinsed twice with dH2O for 5 min each. They were then transferred to a 28% ammonium hydroxide solution (#221228; Sigma‐Aldrich, Belgium) and rotated at room temperature for 30 min. Following two dH2O washes (5 min each), the tissues were immersed in a 15% Kodak Carestream Fixer solution (#P6557; Sigma‐Aldrich, Belgium) at room temperature for 10 min, followed by two 5‐min dH_2_O rinses. Subsequently, after a 60‐min incubation in 28% ammonium hydroxide, the tissues were washed with dH2O and embedded in 3% agar. Slices of 150–300 μm thickness were then prepared. The slices were incubated in a 15% Kodak Carestream Fixer solution, heated at room temperature for 10 min, mounted on glass slides coated with gelatin, air‐dried at room temperature, and covered with glass coverslips using Mowiol (#81381‐50G; Sigma‐Aldrich). Finally, the entire slice was imaged using a Zeiss Axioscope microscope.

The Golgi‐Cox solution comprises a mixture of three components: (A) 5% potassium dichromate (in dH_2_O) (#P5271; Sigma‐Aldrich, Belgium); (B) 5% mercuric chloride (#KK04.1; Carl Roth GmbH, Germany) dissolved in water heated to 60°C; (C) 5% potassium chromate dissolved in water (#HN33.1; Carl Roth GmbH, Germany). Initially, 50 mL of room temperature solution A was mixed in equal parts with 50 mL of room temperature solution B. Subsequently, 40 mL of room temperature solution C was mixed with 100 mL of dH_2_O, and, with continuous stirring, the mixture of solutions A and B was slowly added. After stirring for 2–5 min, the solution was covered with aluminum foil and left at room temperature overnight. The formed red/yellow precipitate was carefully removed, and the remaining supernatant was filtered through a 0.2 μm filter. The prepared solution should be used within 20–30 days.^48^

### 2.12 H&E staining

To preserve the structural integrity of the hippocampal tissue, it was fixed in formaldehyde for 24 h. After fixation, a gradient dehydration process was performed using different concentrations of alcohol (70%, 80%, 90%, 95%, and 100%) for 1 min each to remove water from the tissue. Subsequently, xylene was used for 5 min in each step to make the tissue transparent for easier sectioning and staining. Once the tissue was transparent, it was embedded in paraffin to create 4‐μm thick tissue sections. During the staining process, the conventional H&E method was followed. Following the sealing of the slides, tissue sections were examined under a microscope to observe tissue structure and cell morphology and to facilitate image capture and analysis.

### 2.13 Immunohistochemistry

First, specimen preparation was performed. Mice were anesthetized with an intraperitoneal injection of 0.3% sodium pentobarbital (0.15 mL/10 g), and then cardiac perfusion was performed using 0.9% sodium chloride solution followed by 4% paraformaldehyde solution. After perfusion, brain tissue was removed and fixed in a 4% paraformaldehyde solution for 24 h. Following fixation, the tissue was dehydrated, embedded in paraffin, and cut into 4 μm thick sections for later use. The immunohistochemistry staining procedure started with drying the paraffin sections in a 60°C oven. Deparaffinization was then carried out using xylene, followed by a graded alcohol dehydration process. The sections were then heated in 0.01 M citrate buffer to retrieve antigen activity. After cooling, the sections were rinsed with PBS and blocked with 3% BSA to block nonspecific binding sites. After removing the blocking solution, the sections were incubated overnight at 4°C with specific primary antibodies in PBS. On the next day, the sections were washed with PBS to remove unbound primary antibodies. Subsequently, the sections were incubated with corresponding HRP‐conjugated secondary antibodies for 50 min. After incubation, the sections were washed with PBS again and then subjected to DAB staining. The staining time was controlled under a microscope until positive reactions appeared brownish‐yellow, and then the staining reaction was terminated by washing with PBS. Finally, counterstaining with hematoxylin and coverslipping was performed, followed by microscopic examination and image acquisition for analysis.

### 2.14 Immunofluorescence staining

The brain tissue was placed in a cold 4% paraformaldehyde (PFA‐PBS) solution and fixed overnight. Following dehydration in a PBS solution containing 20% sucrose for two days, the tissue was embedded in OCT compound (Sakura, 4583) and stored at −80°C. Subsequently, frozen sections were obtained using a Leica CM 3050 cryostat. For immunofluorescence staining, brain sections were rehydrated in PBS, permeabilized in 0.5% Triton X‐100 PBS solution for approximately 15 min at room temperature, and then blocked in a solution containing 1% BSA, 2% donkey serum, and 0.3% Triton X‐100 in PBS (blocking buffer) for about 30 min. The primary antibody (diluted 1:100) was applied to the sections in the blocking buffer and incubated overnight at 4°C. The next day, the sections were washed three times with PBS and then incubated with the secondary antibody (Thermofisher Scientific, A10040) for 2 h at room temperature. DAPI was used to stain cell nuclei during the incubation with the secondary antibody (diluted 1:200). Subsequently, the sections were washed three times with PBS, mounted using Fluoromount‐BELG (0100‐BEL 01, Southern Biotech), and stored at 4°C. After immunofluorescence staining, imaging of the sections was performed using laser scanning confocal microscopes (Leica SP5, LSM 780, LSM 800). Primary antibodies used included GFAP (Abcam, ab7260) and Aβ (Santa Cruz Biotechnology, SC‐28365).^49^

### 2.15 TUNEL staining

The apoptotic status of the hippocampal regions in each mouse group was assessed using TUNEL staining with the In Situ Cell Death Detection Kit (Roche, 11684795910, Germany) following the manufacturer's instructions. In brief, 5 μm thick paraffin sections were prepared and immersed in xylene I and II (Sigma‐Aldrich, 296333, USA) for 15 min, followed by dehydration in 100%, 85%, 75% ethanol (Sigma‐Aldrich, 49836, USA), and distilled water for 5 min each for deparaffinization. Subsequently, the sections were incubated with diluted proteinase K (DAKO, Cat.#S3020, USA) at 37°C for 30 min, followed by incubation with permeabilization buffer (Aviva, OOMB00004, USA) at room temperature for 20 min. The sections were then incubated with a mixture of TUNEL reaction solution (TdT:dUTP = 2:29) at 37°C for 2 h, counterstained with DAPI (Sigma‐Aldrich, D9542, USA) for 10 min to stain the cell nuclei, and sealed with anti‐fade mounting medium Fluoroshield™ (Sigma‐Aldrich, F6182, USA). Images of the prepared sections were captured using a NIKON ECLIPSE TI‐SR inverted fluorescence microscope (NIKON, Japan) with excitation wavelengths of 330–380 nm and emission wavelength of 420 nm (for DAPI); and excitation at 465–495 nm and emission at 515–555 nm (for TUNEL). Image analysis and quantification were performed using Image‐Pro Plus 6.0 software (Media Cybernetics, Rockville, MD, USA). The ratio of TUNEL‐positive cells to total cell nuclei (DAPI staining) was used to represent the intensity of TUNEL staining.^50^

### 2.16 Statistical analysis

The bioinformatics analysis was carried out using R software version 4.2.1, and the statistical analysis of experimental data was performed using SPSS software version 25.0. Prior to conducting any tests, the normality of the data distribution was assessed using the Shapiro–Wilk test. *T*‐tests and analysis of variance (ANOVA) were performed for data that satisfied the assumptions of normality and homogeneity of variances. Non‐parametric equivalent methods like the Mann–Whitney *U* test and Wilcoxon signed‐rank test were applied for analysis in cases where the data did not adhere to a normal distribution. Specifically, for differential gene expression analysis, the “limma” package in R 4.2.1 was used to identify differentially expressed genes, with a threshold set at |log_2_(Fold Change)| > 1 and a significance level of *p* < 0.05. Subsequently, the selected differentially expressed genes were subjected to gene set enrichment analysis, utilizing the “ClusterProfiler” package in R 4.2.1 for GO and KEGG pathway analysis, with a significance threshold of *p* < 0.05.

In regard to network analysis, Cytoscape software was employed for the visualization and analysis of the “drug‐component‐target‐disease” network, with the cytoNCA plugin utilized to assess the importance of nodes in the network using BC, CC, and DC algorithms. All statistical analyses of experimental data were performed using SPSS software version 25.0. For data that did not follow a normal distribution, non‐parametric tests such as Kruskal–Wallis Test and Dunn's test were employed for overall and post hoc analysis, while data that followed a normal distribution were analyzed using One‐way ANOVA and Tukey HSD post hoc test. Finally, to provide a clear and visual presentation of the analysis results, GraphPad Prism 8 software was employed for data visualization.

## 3 RESULTS

### 3.1 Pharmacological screening of Ginkgo biloba leaves for candidate genes and chemical components alleviating Alzheimer's disease

**FIGURE 1:**
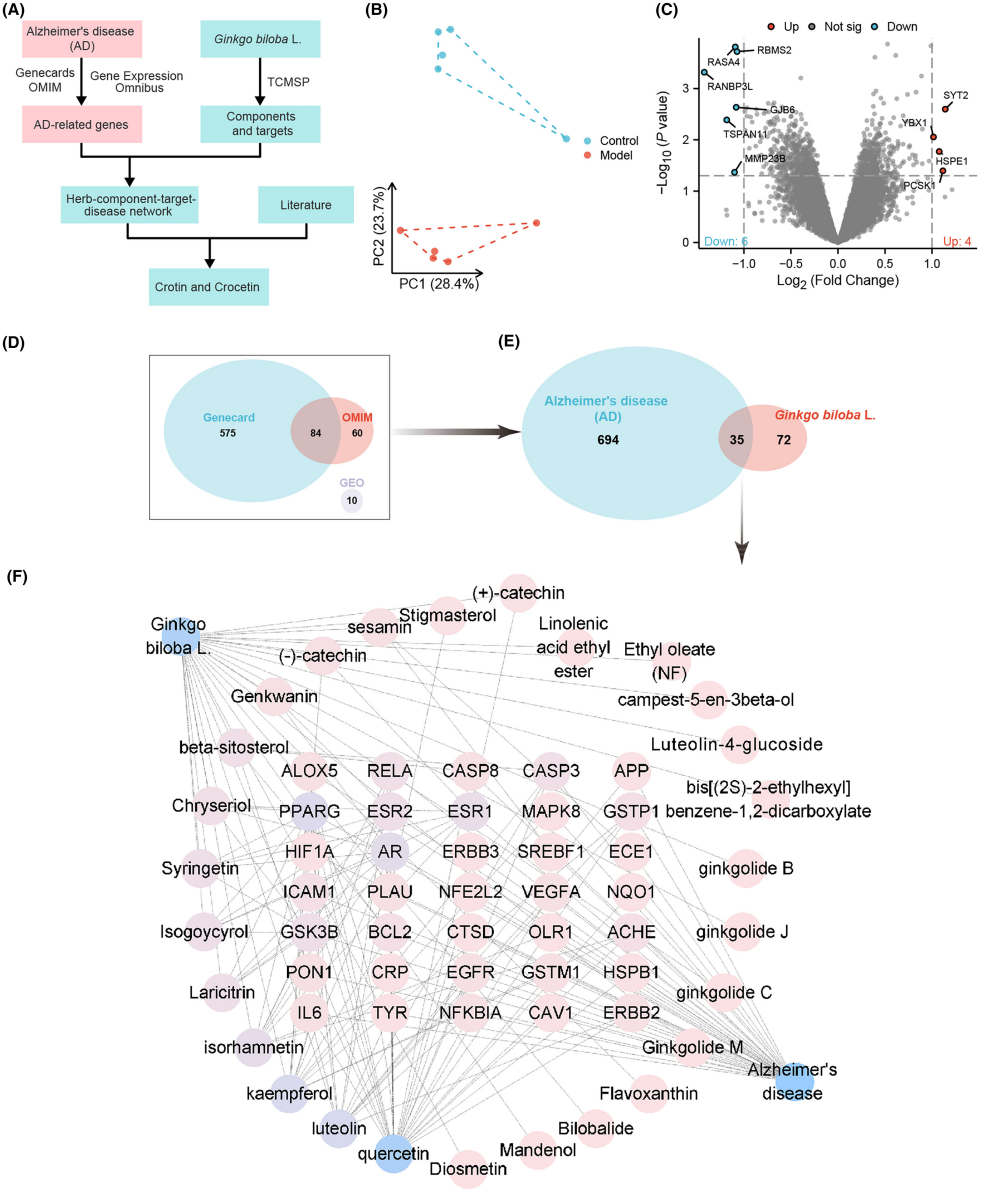
Screening of candidate genes and chemical components in Ginkgo biloba leaves for alleviating Alzheimer's disease. (A) Schematic diagram of the process for pharmacological network screening of candidate genes and chemical components in Ginkgo biloba leaves for alleviating Alzheimer's disease; (B) PCA principal component analysis plot of GSE37263; (C) Volcano plot based on differential expression analysis results in GSE37263; (D) Venn diagram showing the Alzheimer's disease target genes obtained from the Genecard database, OMIM database, and GEO dataset; (E) Venn diagram showing the target genes of Ginkgo biloba leaf effective chemical components and Alzheimer's disease; (F) network interaction diagram of Ginkgo biloba leaves—effective components—target genes—Alzheimer's disease constructed using Cytoscape software, with Ginkgo biloba leaves and Alzheimer's disease located in the upper right and lower left corners, respectively; common target genes of the disease and drugs are displayed in the middle matrix area, while the effective components of Ginkgo biloba leaves are represented by circles. Node size and color intensity indicate the degree value of the node.

AD is a complex neurodegenerative disease with treatment methods and mechanisms still being explored. Ginkgo biloba leaves, as a traditional herbal medicine, have attracted considerable attention due to their potential anti‐AD activity. This study employed network pharmacology methods to screen and analyze the candidate genes and chemical components in Ginkgo biloba leaves that may alleviate AD (Figure 1A) to provide a scientific basis for understanding its mechanism of action.

Based on the GSE37263 dataset, a principal component analysis was performed. The results showed a clear separation between the Control group and the Model group (Figure 1B). Using thresholds of |log_2_(Fold Change)| > 1 and significance *p* < 0.05, 10 differentially expressed target genes were identified (Figure 1C). Additionally, we identified 659 and 144 genes related to AD in GeneCards and OMIM databases, respectively. After removing duplicate target genes from the three screening results, we obtained a total of 729 target genes related to AD (Figure 1D).

Active chemical components of Ginkgo biloba leaves were obtained from the TCMSP database and BATMAN‐TCM database, and these components were screened. The TCMSP database identified 27 active components of Ginkgo biloba leaves. Subsequently, we retrieved the target genes of these 27 chemical components from the TCMSP database. By intersecting these drug targets with disease targets related to AD, a total of 35 candidate genes were discovered (Figure 1E).

Next, we imported the above data into Cytoscape software to construct a network including Ginkgo biloba leaves, active components, target genes, and AD (Figure 1F). Node analysis using the software revealed that quercetin, luteolin, and kaempferol had the highest degree values (i.e., the highest number of connections with other nodes). This suggests that quercetin, luteolin, and kaempferol play important roles in alleviating AD with Ginkgo biloba leaves. Numerous studies have shown that quercetin,^51^ luteolin,^52^, ^53^ and kaempferol^54^ may be effective components in alleviating AD, providing a comprehensive explanation for the clinical use of Ginkgo biloba leaf extract in treating Alzheimer's disease.^55^

In sum, this study successfully employed network pharmacology methods to screen and analyze potential candidate genes and chemical compounds in Ginkgo biloba leaves that may alleviate AD, particularly quercetin, luteolin, and kaempferol. The identification of these components provides a scientific basis for the potential application of Ginkgo biloba leaves in the treatment of AD and contributes to a deeper understanding of its mechanisms of action in AD therapy.

### 3.2 Ginkgo leaf‐regulated candidate genes of Alzheimer's disease: insights from bioinformatics analysis

AD is a complex neurodegenerative disorder with an unclear pathogenesis. To gain a deeper understanding of the key mechanisms underlying AD, this study conducted a comprehensive analysis of candidate genes regulated by ginkgo leaves through bioinformatics methods. We performed GO functional analysis and KEGG pathway analysis on the previously selected 35 candidate genes to reveal their potential roles in AD.

**FIGURE 2:**
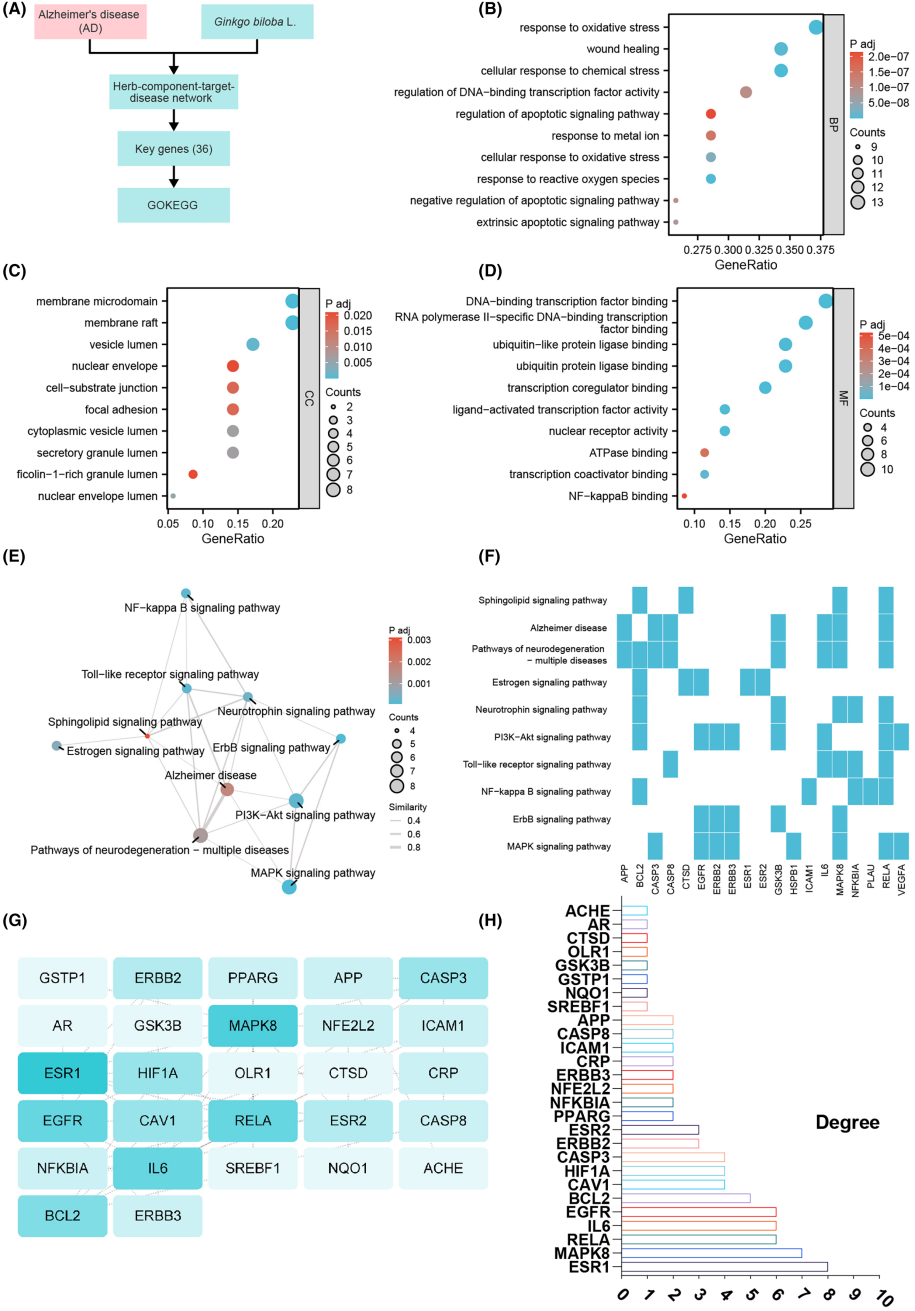
Bioinformatics analysis of Alzheimer's disease candidate genes regulated by Gingko leaf extract and its key signaling pathways. (A) Schematic representation of the bioinformatics analysis workflow, illustrating the analytical steps involved in the potential regulation of AD key signaling pathways by Gingko leaf extract. (B–D) GO analysis results of AD candidate genes possibly regulated by Gingko leaf extract, showing the top 10 results for biological processes (BP), cellular components (CC), and molecular functions (MF), with adjusted *p*‐values. (E) KEGG pathway analysis results of 35 AD candidate genes potentially regulated by Gingko leaf extract, represented by a GOKEGG‐EMAP diagram, with the size of the dots indicating the number of selected genes and the color intensity representing the significance of the enrichment analysis (*p*‐value). (F) KEGG enriched pathways for gene heatmap analysis (G) protein–protein interaction (PPI) network diagram of the 35 AD candidate genes potentially regulated by Gingko leaf extract, constructed using STRING database data and visualized using Cytoscape software. The font size and background color of the genes in the network diagram represent the degree value. (H) Ranking of degree values in the protein–protein interaction network, highlighting the proteins that may play a crucial role in AD.

In the GO functional analysis, we ranked the results according to the standard of *p*.adjust <0.01. The top 10 biological processes (BP) included response to oxidative stress, cellular response to chemical stress, response to reactive oxygen species, and regulation of DNA‐binding transcription factor activity. Regarding cellular components (CC), the major enrichments were found in the membrane raft, membrane microdomain, and vesicle lumen. In terms of molecular functions (MF), enrichments were observed in transcription coregulator binding, DNA‐binding transcription factor binding, RNA polymerase II‐specific DNA‐binding transcription factor binding, and NF‐kappaB binding (Figure 2A–D). The KEGG pathway analysis revealed that the enriched signal pathways mainly involved the MAPK signaling pathway, ErbB signaling pathway, NF‐kappa B signaling pathway, Toll‐like receptor signaling pathway, and PI3K‐Akt signaling pathway (Figure 2E). Notably, the MAPK signaling pathway and PI3K‐Akt signaling pathway exhibited the highest number of enriched genes (Figure 2E). These findings suggest that inflammatory signal pathways associated with PI3K‐Akt signaling and NF‐kappa B signaling may play crucial roles in AD. Further analysis of these enriched KEGG pathways using gene heatmaps demonstrated that RELA was most significantly enriched in these pathways (Figure 2F). Additionally, we imported the 35 candidate genes into the STRING database and constructed a protein–protein interaction (PPI) network (Figure 2G). The analysis revealed that ESR1, MAPK8, RELA, IL6, EGFR, and BCL2 had the highest degree values (Figure 2H), indicating their potential key roles in the occurrence and development of AD.

The bioinformatics analysis conducted in this study revealed the importance of biological processes related to oxidative stress, cellular response to chemical stress, reactive oxygen species response, and DNA‐binding transcription factor activity among the candidate genes regulated by ginkgo leaves in AD. Furthermore, we identified the PI3K‐Akt signaling pathway and NF‐kappa B signaling pathway as potentially playing a central role in the pathogenesis of AD.

### 3.3 Effects of Ginkgo biloba leaf extract on cognitive function and neuronal morphology in mice

This study aims to investigate the impact of Ginkgo biloba leaf extract (GBE) on cognitive function and neuronal morphology in mice through a series of experimental interventions. By analyzing the changes in body weight, behavior in an open field test, learning and memory abilities in the Morris water maze test, and morphological changes in hippocampal neurons before and after the experimental interventions, this study seeks to gain a deeper understanding of the potential neuroprotective effects of GBE.

**FIGURE 3:**
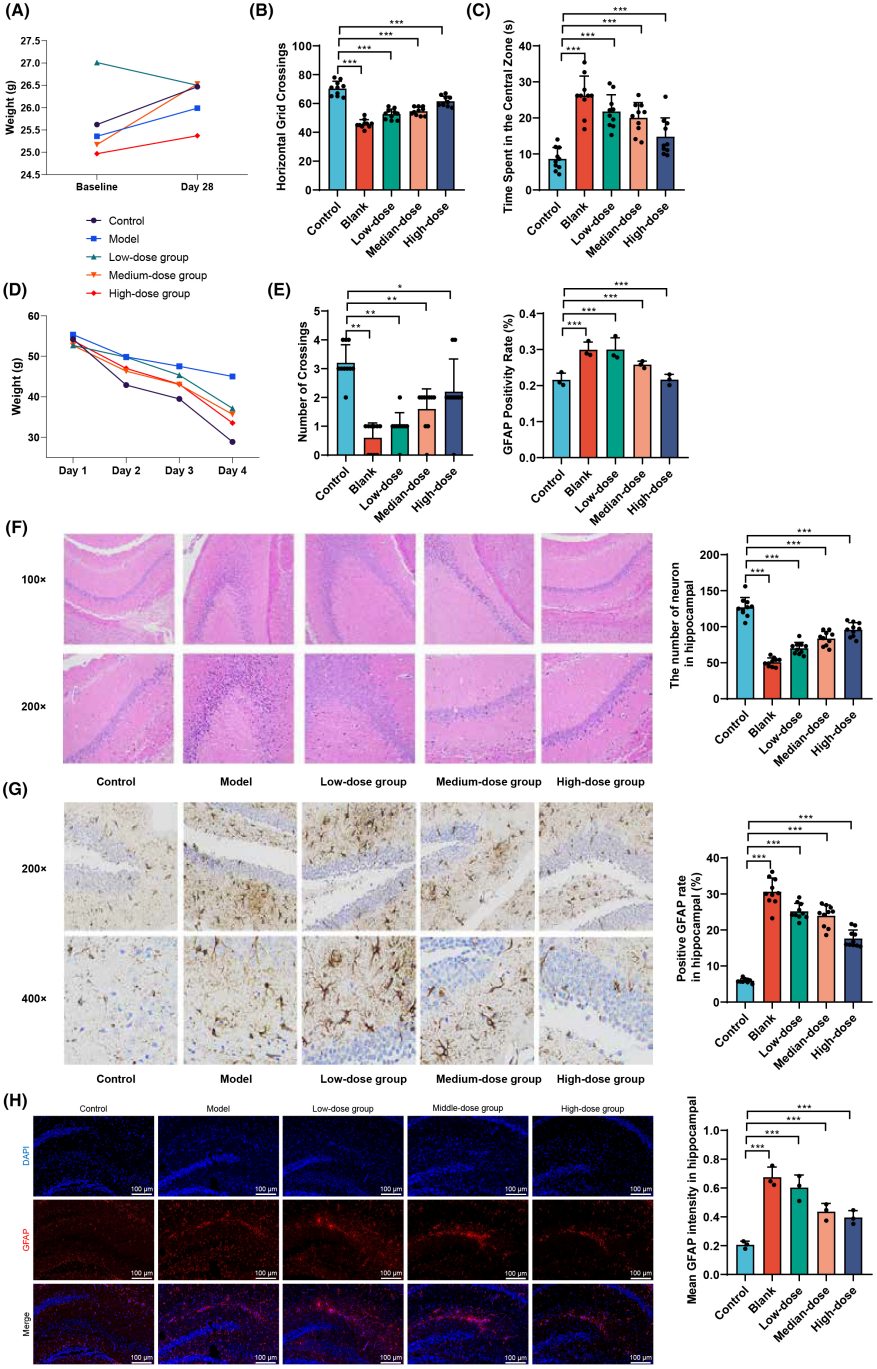
Effects of Ginkgo biloba leaf extract (GBE) on mouse behavior and neuronal morphology. (A) Comparison of body weight among the different groups of mice before and after experimental intervention. (B, C) Horizontal entries and central grid dwell time of mice in the open field test. (D, E) Escape latency and platform crossings of mice in the Morris water maze test. (F) H&E staining observation and quantitative analysis of the hippocampal region in each group of mice (×20) demonstrate morphological changes of nerve cells and neuron counts. (G) Immunopositive expression of GFAP protein in the hippocampal region of mice in each group. (H) Immunofluorescent staining of GFAP protein in the hippocampal region of mice in each group. *p < 0.05; **p < 0.01; ***p < 0.001; ns indicates no statistical significance (*n* = 10).

A two‐factor unidirectional repeated measures analysis of variance showed no statistically significant differences in body weight among the mice groups before and after the experimental interventions (Table 1, Figure 3A, *p* > 0.05). In the open field test (Figure 3B,C), there were significant differences in the horizontal grid crossings among the groups (*p* < 0.05). Compared to the Control group, all GBE treatment groups had lower numbers of horizontal‐grid crossings, while compared to the Model group, different dosages of GBE treatment groups had higher numbers of horizontal‐grid crossings (*p* < 0.05). Similarly, there were significant differences in the central‐grid dwelling time (*p* < 0.05). Compared to the Control group, all groups had higher central‐grid dwelling times, while compared to the Model group, the medium and high‐dosage GBE treatment groups had lower central‐grid dwelling times (*p* < 0.05).

**TABLE 1:** qRT‐PCR primer sequences.

Name	Primer sequences
PI3K	F: 5′‐ACACCACGGTTTGGACTATGG‐3′
	R: 5′‐GGCTACAGTAGTGGGCTTGG‐3′
AKT	F: 5′‐ATGAACGACGTAGCCATTGTG‐3′
	R: 5′‐TTGTAGCCAATAAAGGTGCCAT‐3′
NF‐κB	F: 5′‐GCCAAAGAAGGACACGAC‐3′
	R: 5′‐ATGTCTGCTGCTGCTGGT‐3′
β‐Actin	F: 5′‐GGCTGTATTCCCCTCCATCG‐3′
	R: 5′‐CCAGTTGGTAACAATGCCATGT‐3′

The Morris water maze test (Figure 3D,E, Table 2) showed no statistically significant differences in escape latency duration among the mice groups from day 1 to day 3 (*p* > 0.05), but on day 4, there were significant differences (*F* = 14.909, *p* < 0.01). The Model group had a longer escape latency duration compared to the Control group (*p* < 0.05), while the high‐dosage GBE treatment group had a shorter escape latency duration compared to the Model group (*p* < 0.05). In terms of spatial exploration, there were significant differences in the number of platform crossings among the groups (*F* = 33.180, *p* < 0.001). Compared to the Control group, the Model group and GBE low and medium dosage groups had lower numbers of platform crossings (*p* < 0.05), while compared to the Model group, the high‐dose GBE treatment group had higher numbers of platform crossings (*p* < 0.05).

**TABLE 2:** Comparison of body weight in mice before and after experimental intervention.

Group	Time	Weight (g)
Control	Baseline	25.62 ± 1.75
Model	Baseline	25.36 ± 1.89
Low‐dose group	Baseline	27.01 ± 2.14
Medium‐dose group	Baseline	25.17 ± 2.11
High‐dose group	Baseline	24.97 ± 2.67
Control	Day 28	26.47 ± 2.75
Model	Day 28	25.99 ± 0.97
Low‐dose group	Day 28	26.50 ± 1.97
Medium‐dose group	Day 28	26.53 ± 3.47
High‐dose group	Day 28	25.37 ± 1.29

*Note*: Number of mice per group is 10 (*n* = 10). Data are presented as mean ± standard deviation.

Hematoxylin and eosin staining (Figure 3F) showed that the Control group had numerous intact hippocampal neurons with no deformation, a tightly packed cell layer, normal size, and intact structure. The Model group had incomplete neuronal morphology with evident deformed cells, nuclear condensation, reduced pyramidal cells, decreased granule cell numbers, increased intercellular gaps, and disordered arrangement. The low and medium‐dosage GBE treatment groups had relatively intact cell morphology with slight nuclear condensation and decreased numbers. The high‐dosage GBE treatment group had a greater number of cells, with a few cells showing morphological deformities, overall close arrangement, and intact structure. Quantitative statistical results indicate a significant increase in the number of hippocampal neurons in low, medium, and high‐dose GBE groups compared to the Model group. Immunohistochemistry staining for GFAP protein (Figure 3G) showed that compared to the Control group, the Model group and low‐dosage GBE group had a significantly increased expression of GFAP (*p* < 0.01). Compared to the Model group, the high‐dosage GBE treatment group had a significantly decreased expression of GFAP (*p* < 0.01). Simultaneous use of immunofluorescence labeling to detect GFAP protein in microglial cells revealed a significant increase in GFAP fluorescence intensity in the Model group compared to the Control group (*p* < 0.01), as shown in Figure 3H. Furthermore, the high and medium doses of GBE led to a significant decrease in GFAP fluorescence intensity compared to the Model group (*p* < 0.01).

In summary, the results of this study demonstrate that GBE has no significant effect on the body weight of mice but significantly improves the number of horizontal‐grid crossings and central‐grid dwelling time in the open field test, particularly in the high dosage group. The Morris water maze test further confirms the improvement of learning and memory abilities in the high‐dosage GBE group. Hematoxylin and eosin staining and GFAP protein expression analysis revealed the protective effect of GBE on the morphology of hippocampal neurons and abnormal activation of microglial cells, especially in the high‐dosage group. These findings provide strong experimental support for the potential application of GBE in neuroprotection and improvement of cognitive function.

### 3.4 The protective effects of GBE on hippocampal neurons in APP/PS1 mice and its inhibition of amyloid pathology

**FIGURE 4:**
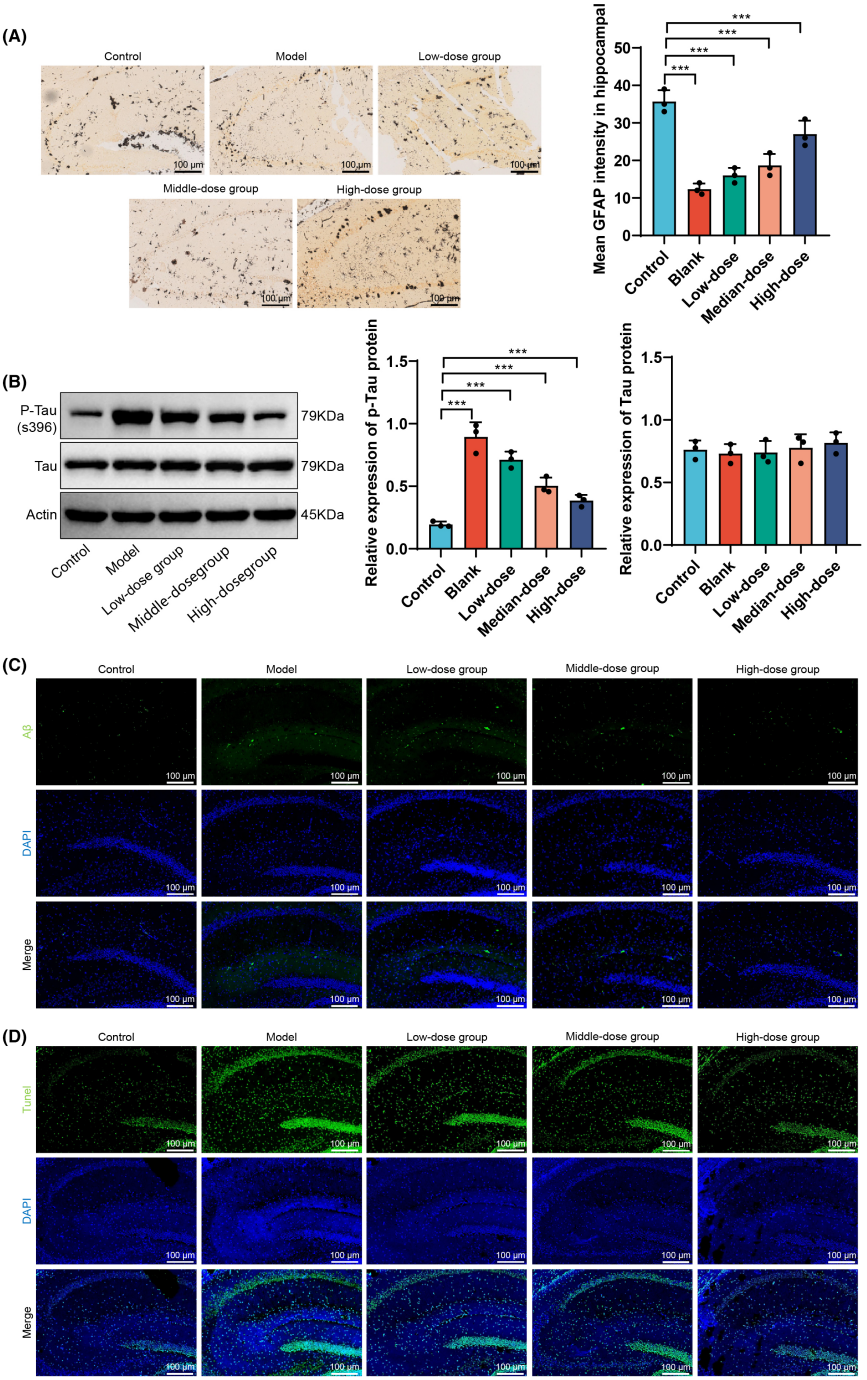
The protective effect of GBE on mouse hippocampal neurons and the inhibition of amyloid pathological changes. (A) Dendritic spine morphology and quantity of mouse hippocampal neurons in different groups. (B) Western blot analysis shows the expression levels of p‐Tau and Tau proteins in mouse groups. (C) Expression of amyloid protein in mouse hippocampus of different groups. (D) Expression of TUNEL‐positive cells in the hippocampus of mice in different groups. ***p < 0.001; ns indicates no statistical significance (*n* = 10).

In this study, Golgi silver staining was employed to examine the morphology and quantity of dendritic spines in the hippocampal neurons of mice from different groups following GBE intervention (Figure 4A). The results indicate a significant reduction in the number of dendritic spines in the model group compared to the control group, while the high‐dose GBE group showed the most prominent increase in dendritic spine quantity compared to the model group. Additionally, immunofluorescent staining using amyloid protein antibodies was utilized to assess amyloid protein expression in the hippocampus of mice in different groups, and protein immunoblotting was employed to measure the expression levels of phosphorylated Tau protein (Figure 4B,C). The findings reveal a significant increase in amyloid protein and phosphorylated Tau protein expression in the model group compared to the control group, whereas the high‐dose GBE group exhibited the most significant decrease in amyloid protein and phosphorylated Tau protein levels compared to the model group. These results suggest that GBE intervention could alleviate amyloid pathology in the brains of APP/PS1 mice. Lastly, TUNEL staining was utilized to detect TUNEL‐positive cells in the hippocampus of mice from different groups following GBE intervention (Figure 4D). The results showed a significant increase in TUNEL‐positive cell expression in the model group compared to the control group, with the high‐dose GBE group exhibiting the most notable decrease in TUNEL‐positive cells compared to the model group. Overall, the study results indicate that GBE intervention can mitigate dendritic spine damage, alleviate amyloid pathology, and suppress neuronal apoptosis in the brains of APP/PS1 mice.

### 3.5 The effects of Ginkgo biloba leaf extract on the protein and mRNA expression of the PI3K/AKT/NF‐κB signaling pathway

Based on the results of bioinformatics analysis, the aim of this study was to investigate the effects of GBE on the protein and mRNA expression of key components in the PI3K/AKT/NF‐κB signaling pathway. The modulation of GBE on the protein and mRNA expression levels of PI3K, AKT, and NF‐κB in a mouse model was extensively analyzed using Western blot and qRT‐PCR techniques qRT‐PCR Primer Sequences are shown in Table 3. The goal was to elucidate the potential mechanisms of GBE in neuroprotection and anti‐inflammatory effects.

**TABLE 3:** Comparison of Morris water maze test results among different mouse groups.

Group	Escape latency (is)				Number of crossings
	Day 1	Day 2	Day 3	Day 4	
Control	54.24 ± 2.91	42.88 ± 8.23	39.48 ± 7.14	28.86 ± 8.31	3.20 ± 0.63
Model	55.34 ± 3.26	49.85 ± 8.24	47.53 ± 5.42	45.02 ± 8.79^a^	0.60 ± 0.52^a^
Low‐dose group	52.68 ± 5.30	49.78 ± 8.32	45.34 ± 7.61	37.15 ± 8.02	1.00 ± 0.47^a^
Medium‐dose group	52.67 ± 7.05	46.34 ± 7.55	43.01 ± 6.23	35.66 ± 9.00	1.60 ± 0.70^a^
High‐dose group	53.87 ± 9.95	47.02 ± 8.11	43.11 ± 6.34	33.54 ± 6.13^b^	2.20 ± 1.14^b^
*F*	2.6749	5.3078	2.0738	14.909	33.180
*p*	0.6136	0.2571	0.1001	0.0049	0.000

*Note*: Compared to the control group, ^a^ *p* <0.05; compared to the blank group, ^b^ *p* <0.05. Data are presented as mean ± standard deviation.

**FIGURE 5:**
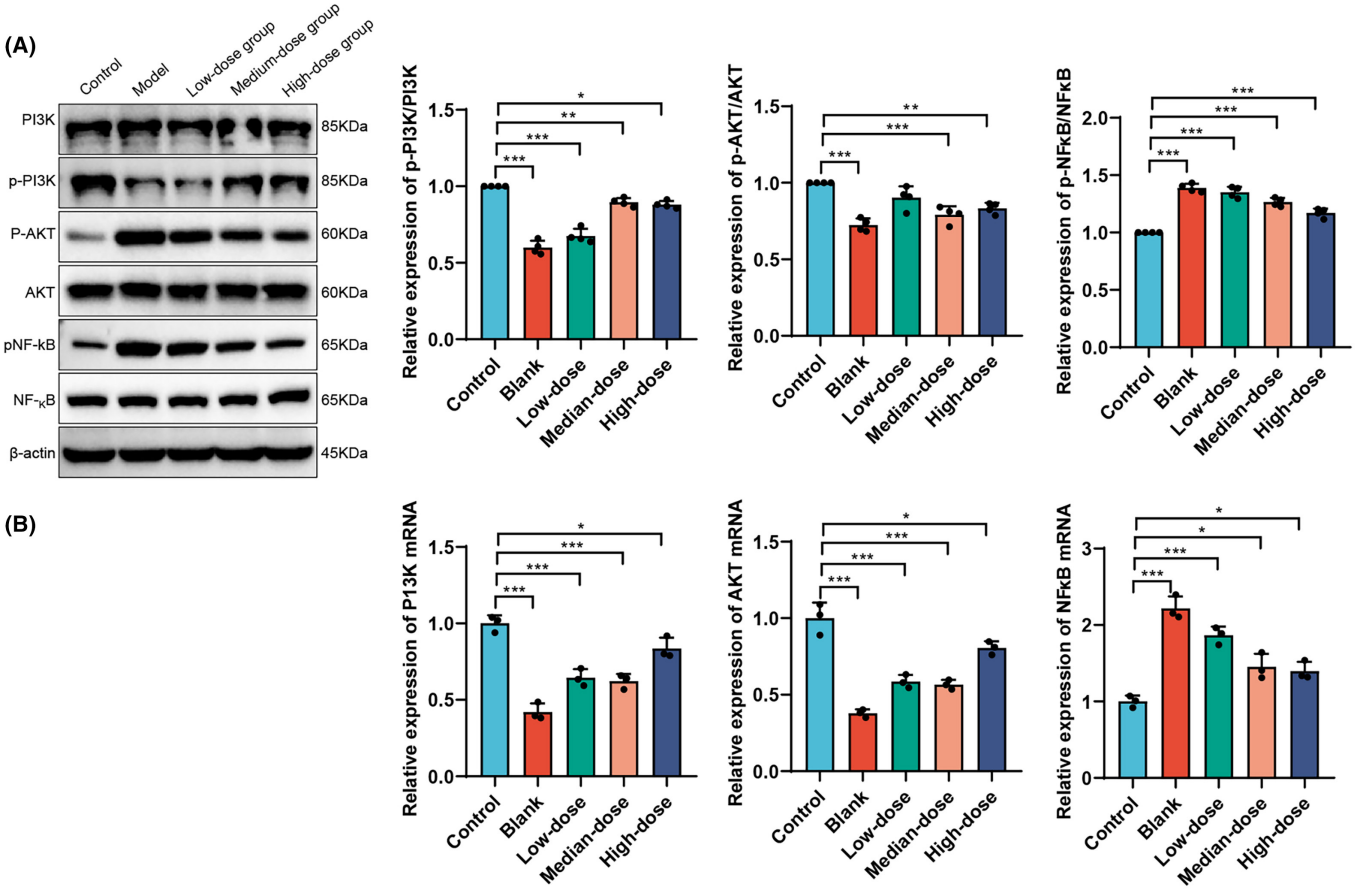
Effects of Gingko biloba leaf extract on the protein and mRNA expression of the PI3K/AKT/NF‐κB signaling pathway. (A) Western blot analysis shows the expression levels of PI3K, p‐PI3K, AKT, p‐AKT, NF‐κB, and p‐NF‐κB proteins in each group of mice. (B) qRT‐PCR analysis shows the expression levels of PI3K, AKT, and NF‐κB mRNA in each group of mice. *p < 0.05; **p < 0.01; ***p < 0.001 (*n* = 10).

The Western blot results (Figure 5A) demonstrated a significant decrease in the ratio of p‐PI3K/PI3K protein expression in the Model group compared to the Control group (*p* < 0.05). However, treatment with GBE in the medium and high dose groups significantly increased the expression levels of p‐PI3K protein compared to the Model group (*p* < 0.05). There was no significant difference in the protein expression levels of p‐AKT between the Model and Control groups (*p* > 0.05). Yet, compared to the Model group, the expression levels of p‐AKT protein were significantly elevated in the low and high‐dose GBE groups (*p* < 0.05). The protein expression levels of p‐NF‐κB significantly increased in the Model group (*p* < 0.05), and compared to the Model group, there was a significant decrease in the expression levels of p‐NF‐κB protein in the medium and high dose GBE groups (*p* < 0.05).

The qRT‐PCR results (Figure 5B) showed a significant decrease in the mRNA expression levels of PI3K and AKT in the Model group, GBE low‐dose group, medium‐dose group, and high‐dose group compared to the Control group (*p* < 0.05). Compared to the Model group, there was a significant increase in the mRNA expression levels of PI3K and AKT in the GBE low‐dose group and GBE high‐dose group (*p* < 0.05). The mRNA expression level of NF‐κB significantly increased in the Model group, GBE low‐dose group, medium‐dose group, and high‐dose group (*p* < 0.05). Compared to the Model group, there was a significant decrease in the mRNA expression levels of NF‐κB in the GBE low‐dose group, medium‐dose group, and high‐dose group (*p* < 0.05).

In summary, GBE exerted significant modulation on the protein and mRNA expression of the PI3K/AKT/NF‐κB signaling pathway. GBE was able to regulate the protein and mRNA expression of PI3K, AKT, and NF‐κB to varying degrees, with a more pronounced effect observed in the medium and high‐dose groups. These results suggest that GBE may exert its neuroprotective and anti‐inflammatory effects by regulating the PI3K/AKT/NF‐κB signaling pathway, providing experimental evidence for its potential application in the treatment of neurodegenerative diseases.

### 3.6 The influence of Ginkgo biloba leaf extract on the levels of the inflammatory factors IL‐1β, TNF‐α, and IL‐6 in mouse serum

This study further explores the impact of GBE on the levels of key inflammatory factors IL‐1β, TNF‐α, and IL‐6 in mouse serum. Utilizing ELISA technology, this section of the investigation aims to evaluate the regulatory effects of GBE on the inflammatory response, thereby providing a deeper understanding of its potential applications in the fields of anti‐inflammation and neuroprotection.

**FIGURE 6:**
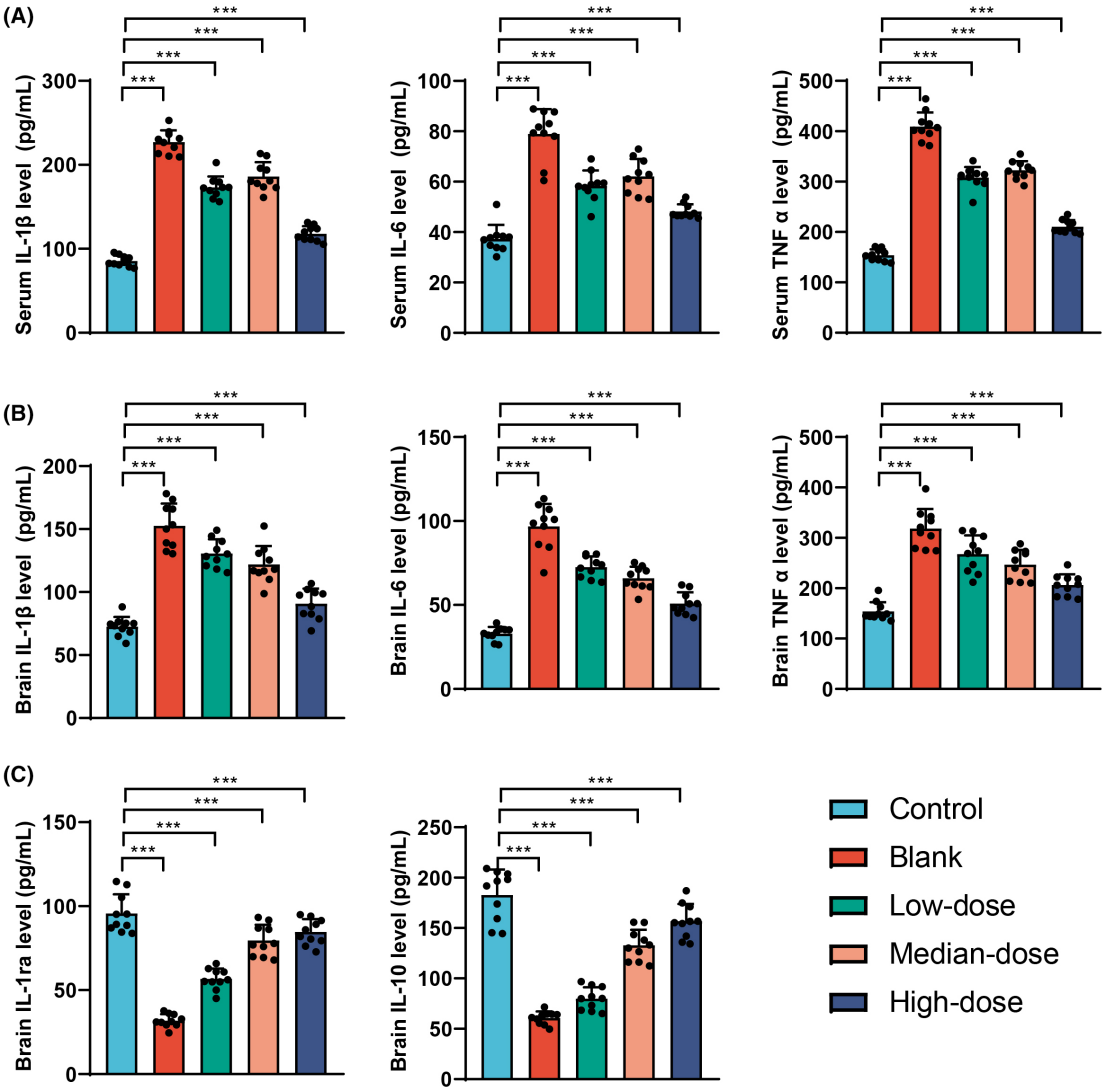
Influence of Ginkgo biloba leaf extract on the levels of inflammatory cytokines in mouse serum. (A) Detection of inflammatory factor levels of IL‐1β, TNF‐α, and IL‐6 in the serum of mice in each group using ELISA technique. (B) Detection of inflammatory factor levels of IL‐1β, TNF‐α, and IL‐6 in the brains of mice in each group using ELISA technique. (C) Detection of anti‐inflammatory factor levels of IL‐1ra and IL‐10 in the brains of mice in each group using ELISA technique. ***p < 0.001 (*n* = 10).

The results obtained from ELISA testing (Figure 6A) reveal that, compared to the Control group, the Model group and the groups treated with low, medium, and high doses of GBE demonstrated significant increases in IL‐1β, TNF‐α and IL‐6 levels in mouse serum, with statistical significance (*p* < 0.01). Compared with the Model group, the groups treated with low, medium, and high doses of GBE exhibited decreases in IL‐1β and TNF‐α levels in mouse serum, demonstrating statistical significance (*p* < 0.001). Notably, the group treated with the high dose of GBE also showed a significant decrease in IL‐6 levels in mouse serum, with statistical significance (*p* < 0.001).

The ELISA assay was used to detect the levels of inflammatory factors IL‐1β, TNF‐α, and IL‐6 in the brains of mice (Figure 6B). The results revealed a significant increase in the levels of IL‐1β, TNF‐α, and IL‐6 in the model group compared to the control group. Following intervention with GBE, the levels of IL‐1β, TNF‐α, and IL‐6 in the brains of mice decreased in the low‐dose group, medium‐dose group, and high‐dose group, with statistically significant differences (*p* < 0.001). Additionally, the expression of anti‐inflammatory factors IL‐1ra and IL‐10 in the brains of mice was assessed using ELISA (Figure 6C). The findings indicated a significant decrease in the levels of IL‐1ra and IL‐10 in the model group compared to the control group. Following GBE intervention, the levels of IL‐1ra and IL‐10 in the brains of mice in the low‐dose group, medium‐dose group, and high‐dose group all showed a significant increase compared to the model group.

In conclusion, Ginkgo biloba leaf extract significantly modulates the levels of inflammatory factors in the serum and brain of mice. Particularly in the high‐dose group, there was a more pronounced reduction in the levels of the pro‐inflammatory factors IL‐1β, TNF‐α, and IL‐6, while simultaneously enhancing the levels of anti‐inflammatory factors in the brain. These findings suggest that GBE has a significant anti‐inflammatory effect, possibly exerting its neuroprotective role by reducing the release of inflammatory factors.

## 4 DISCUSSION

Currently, while there are various new drugs under development for the treatment of AD, their efficacy is generally unsatisfactory.^56^ Current drug treatments mainly focus on slowing down disease progression and controlling certain cognitive and behavioral symptoms, rather than preventing or reversing the disease.^57^, ^58^ Therefore, researchers are striving to find more effective treatment methods and prevention strategies in order to eventually find a cure for AD.

Herbal medicine has shown potential benefits in AD treatment.^59^, ^60^, ^61^ Studies have shown that natural compounds such as curcumin in turmeric, quercetin flavonoids in mulberry, and resveratrol in grapes exhibit neuroprotective effects in cellular and animal models of AD.^62^, ^63^ These research findings provide scientific evidence for the use of natural herbal ingredients as AD treatment strategies.^59^, ^64^, ^65^ In particular, Ginkgo biloba extract (GBE) and its terpenoid compounds have been proven to exhibit neuroprotective effects in neurodegenerative diseases, opening up new perspectives for AD treatment.^66^

In neurodegenerative disease models, GBE has shown significant anti‐inflammatory, antioxidant, and anti‐apoptotic effects.^31^, ^67^ GBE can increase the expression of SIRT‐1, inhibit NF‐kB activity, and upregulate heme oxygenase‐1 (HO‐1) and anti‐apoptotic protein expression while downregulating pro‐apoptotic protein expression. GBE can also improve mitochondrial dysfunction and protect SH‐SY5Y cells from the toxicity of reactive oxygen species (ROS).^68^ In an Aβ25‐35‐induced hippocampal neuron model, GBE can upregulate endogenous neurotrophic factors and effectively inhibit cell apoptosis. These mechanisms of action provide important molecular‐level evidence for the application of GBE in AD treatment.^69^, ^70^, ^71^

This study found that GBE significantly improves cognitive function in APP/PS1‐aged mice, protects neurons, and effectively inhibits the activation of reactive astrocytes (ACs). Additionally, GBE reduces neuroinflammatory response by regulating the PI3K/AKT/NF‐κB signaling pathway. These findings are consistent with previous research, but this study further explores the multiple neuroprotective mechanisms of GBE. In transgenic mice, the elevated peripheral cytokine levels may be attributed to systemic inflammatory responses or peripheral immune system activation.^9^, ^11^ Neuroinflammation caused by amyloid beta or oxidative stress can indeed increase proinflammatory cytokines in brain tissue, and the raised circulating peripheral cytokines may be attributed to the interplay between central and peripheral immune systems. Research has shown that central nervous system inflammation can lead to peripheral immune system activation through the disruption of the blood–brain barrier or the diffusion of inflammatory mediators.^12^ Additionally, peripheral immune cells can influence central nervous system inflammation by producing proinflammatory cytokines.^13^ The mechanism of action of GBE may involve modulation of both central and peripheral immune cells, manifesting as direct inhibition of central nervous system inflammation and regulation of peripheral immune cell activity. For example, behavioral tests such as open field and Morris water maze experiments further confirm the effectiveness of GBE in improving cognitive function in APP/PS1 mice.

The use of GBE in AD treatment demonstrates the potential of herbal medicine as a low‐side‐effect treatment option. Studies have found that combining GBE with other treatment measures may produce better therapeutic effects.^72^, ^73^, ^74^ However, the clinical application of GBE still faces numerous challenges, including determining effective and safe dosages, evaluating long‐term treatment effects, and potential drug interactions.^46^, ^75^ Therefore, despite the potential shown by GBE in AD treatment, its effectiveness and safety in clinical application require further research. Although this study provides promising results, there are limitations. First, the research was conducted only in animal models, and data from human clinical trials are lacking. Therefore, the effects and safety of GBE in human AD patients still need further investigation. Second, the GBE components used in the study are complex, and the specific effective components and mechanisms of action still need to be clarified. Future research should focus on the isolation and identification of these components, as well as clinical trials in AD patients at different stages. Additionally, exploring the combined treatment effects of GBE and existing AD drugs is also an important direction for future research.

The core contribution of this study lies in providing experimental data supporting the application of Ginkgo biloba extract (GBE) in the treatment of AD. We utilized network pharmacology and bioinformatics approaches to investigate the key chemical components in GBE, such as quercetin, luteolin, and kaempferol, which may exert therapeutic effects by targeting the PI3K/AKT/NF‐κB signaling pathway. This important finding provides a valuable scientific basis for the development of novel therapeutic strategies targeting AD. From a clinical perspective, GBE, as a natural medicine, demonstrates potential as a safe and effective treatment option for AD, particularly suitable for patients seeking alternative traditional drug treatments.

Furthermore, this study also presents methodological innovations. We selected APP/PS1 aging mice as the model for AD research, which, compared to traditional drug‐induced models, better reflects the natural, pathological process of AD and provides conditions for a more accurate simulation of the disease's pathological features and progression. The study focused on astrocytes (ACs), neuroinflammation, and the PI3K/AKT/NF‐κB signaling pathway, which play critical roles in cell metabolism, proliferation, survival, and growth in AD. The pathogenesis of AD is closely related to brain insulin resistance, where abnormal glucose utilization can lead to neurodegenerative changes.^76^ Inhibition of GLUT can increase amyloid deposition in the brain.^77^ The PI3K/AKT pathway, as a crucial regulator of brain insulin metabolism, is also involved in the development of AD and amyloid deposition in the brain. Upregulating brain PI3K/AKT expression through intracerebral insulin injection can improve cognitive function and promote neurogenesis in AD model mice, consistent with our research findings.^78^ Activation of NF‐kB is a key factor in the pathologic deposition of amyloid in AD. Excessive NF‐kB activation can impair microglial function and reduce their capacity to clear amyloid deposits.^79^, ^80^ Similarly, our study demonstrates that GBE can alleviate microglial dysfunction and enhance amyloid clearance by inhibiting NF‐kB activation, supporting these findings. Moreover, the study demonstrated the mediating role of inflammation factors between GFAP and cognitive function in a clinical aspect and preliminarily confirmed the effective inhibition of reactive ACs activation and alleviation of neuroinflammation by GBE in the experimental section. These achievements not only enrich our understanding of GBE as a traditional Chinese medicine preparation in the treatment of AD but also provide directions for future clinical research and drug development.

## 5 LIMITATIONS

Our study has several limitations that warrant consideration. Firstly, the reliance on mouse models for investigating the effects of GBE on AD may not fully capture the complexity of AD in humans. Secondly, the precise dosing regimen of GBE in our experiments is not specified, emphasizing the need to determine optimal doses for potential clinical applications. Additionally, while we suggest that GBE may modulate the PI3K/AKT/NF‐κB signaling pathway, a more comprehensive mechanistic understanding is required. The lack of data from human clinical trials, short‐term focus, and the potential for publication bias are also limitations that need to be acknowledged. Finally, any potential conflicts of interest should be transparently disclosed.

In conclusion, this study delved into the therapeutic potential of Ginkgo biloba extract (GBE) for AD. The research findings indicate that GBE can significantly enhance cognitive function in AD model mice, protect nerve cells, and alleviate amyloid beta pathology. This effect may be achieved through the modulation of the PI3K/AKT/NF‐κB signaling pathway, which plays a crucial role in the pathogenesis of AD. The regulatory effect of GBE on this pathway is manifested in the changes in protein and mRNA expression levels. Additionally, GBE can reduce the levels of inflammatory factors in the blood and brain while increasing anti‐inflammatory factors. These discoveries reveal that GBE might combat AD by alleviating neuroinflammation and regulating key signaling pathways.

## 6 CONCLUSIONS

In summary, this study thoroughly explores the therapeutic potential of Ginkgo biloba extract (GBE) in AD. The results demonstrate that GBE significantly improves cognitive function and neuronal morphology in an AD mouse model. This effect may be achieved through the regulation of the PI3K/AKT/NF‐κB signaling pathway, which plays a key role in the pathogenesis of AD. The regulatory effect of GBE on this pathway is manifested through changes in protein and mRNA expression levels, and GBE also reduces the levels of inflammatory factors in the serum. These findings reveal that GBE may counteract AD by alleviating neuroinflammation and modulating key signaling pathways.

## AUTHOR CONTRIBUTIONS

Cheng Zhu led the experiment design and drafted the manuscript. Jie Liu assisted in experimental procedures and data collection. Jixin Lin contributed to the research design and methodology. Jiaxi Xu focused on data interpretation, particularly in psychiatric aspects. Enyan Yu, the corresponding author, coordinated the research, interpreted the results, and finalized the manuscript.

## FUNDING INFORMATION

This study was supported by the Zhejiang Provincial Key R&D Program (Project No. 2021C03106).

## CONFLICT OF INTEREST STATEMENT

The author declares no conflict of interest.

## Supporting information


Appendix S1.

## Data Availability

The datasets used and analyzed during the current study are available from the corresponding author upon reasonable request.
