# Supplementary figures and images for: Investigating the effects of Ginkgo biloba leaf extract on cognitive function in Alzheimer's disease
Source: CNS Neurosci Ther. 2024 Sep 5;30(9):e14914. doi: 10.1111/cns.14914 (PMC11377177; doi:10.1111/cns.14914)

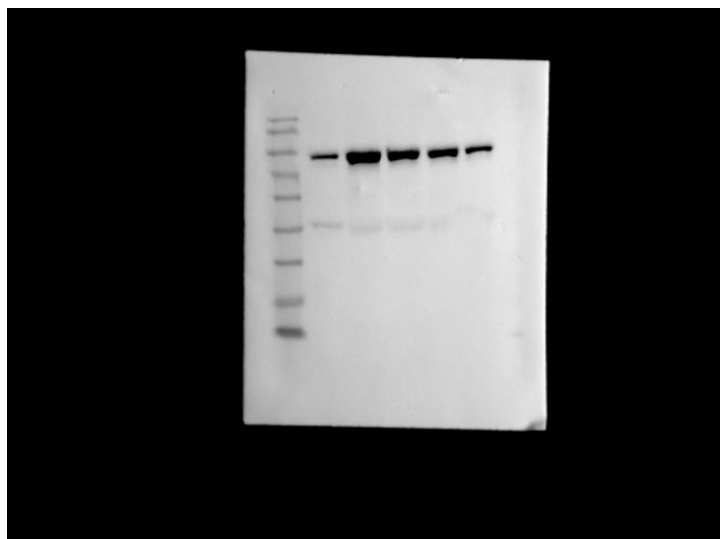

Figure4B-1

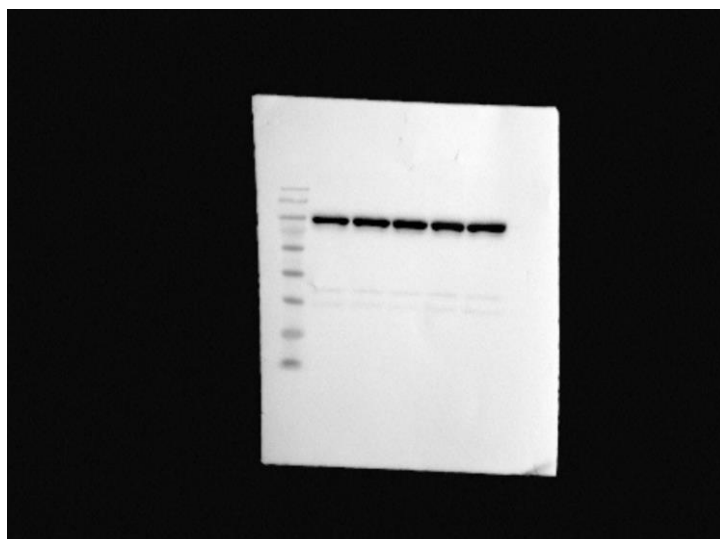

Figure4B-2

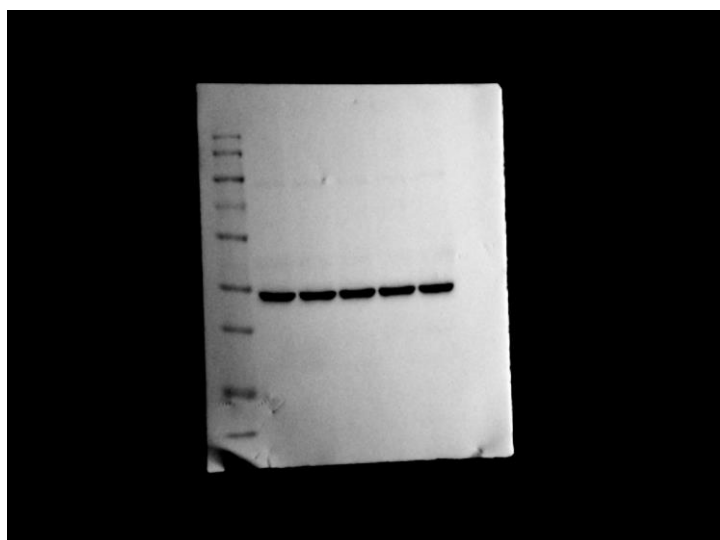

Figure4B-3

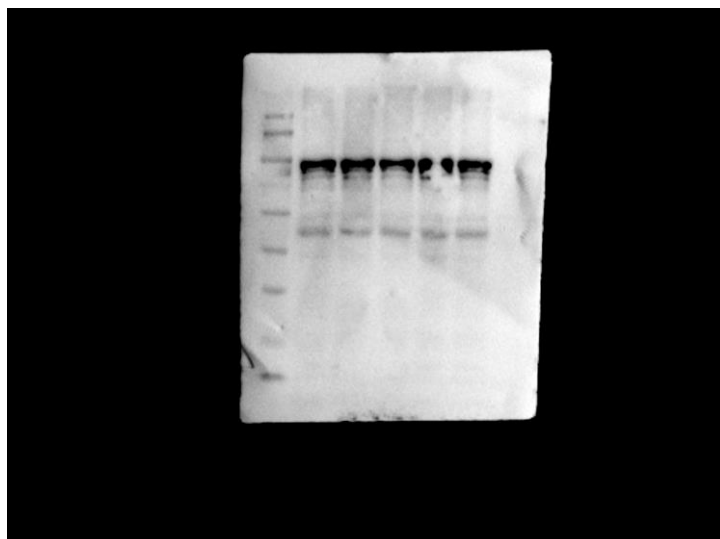

Figure5A-1

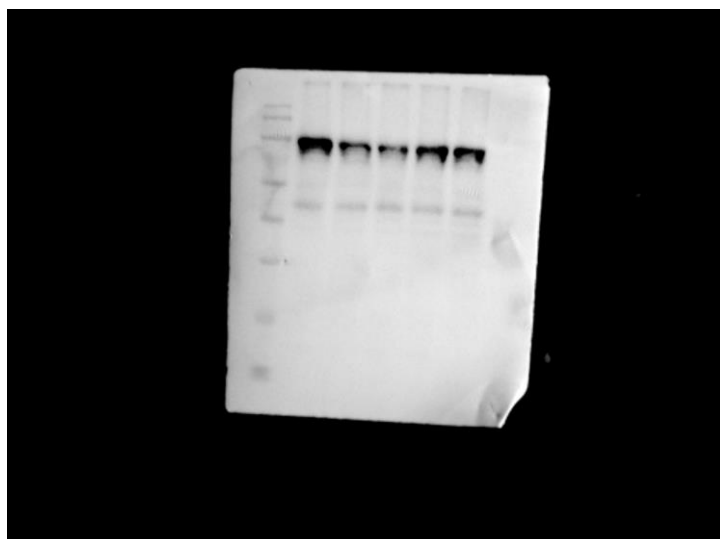

Figure5A-2

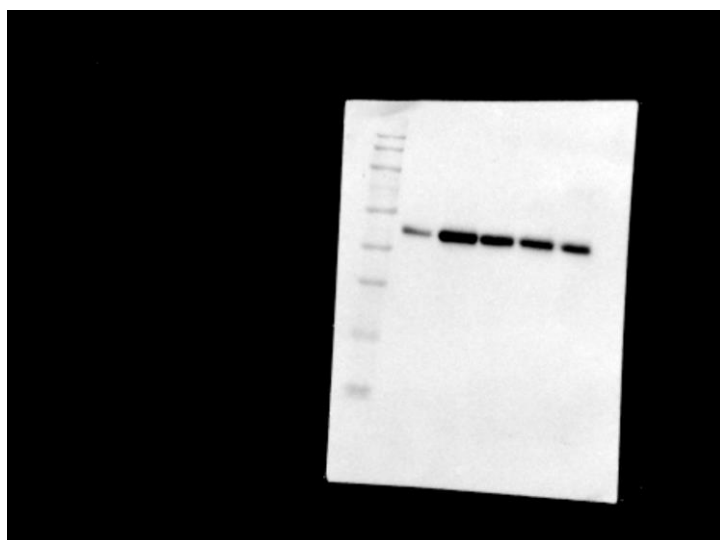

Figure5A-3

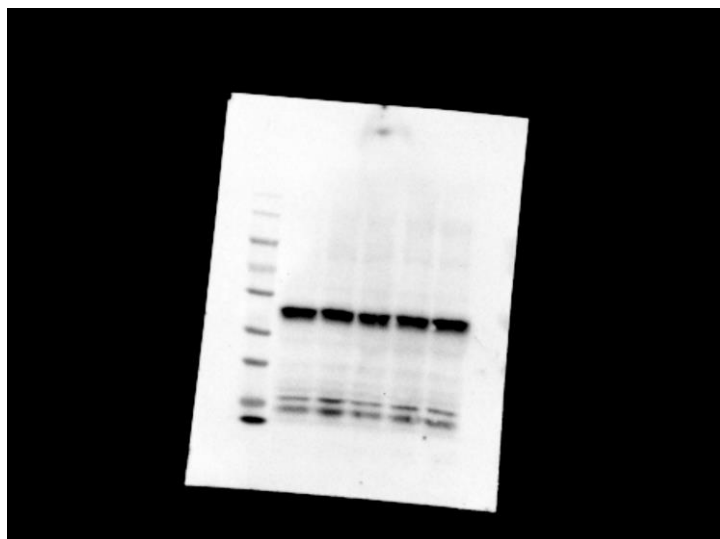

Figure5A-4

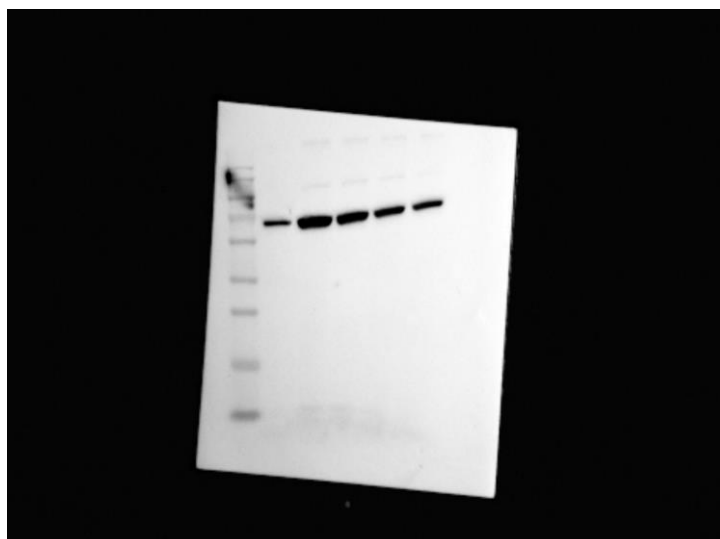

Figure5A-5

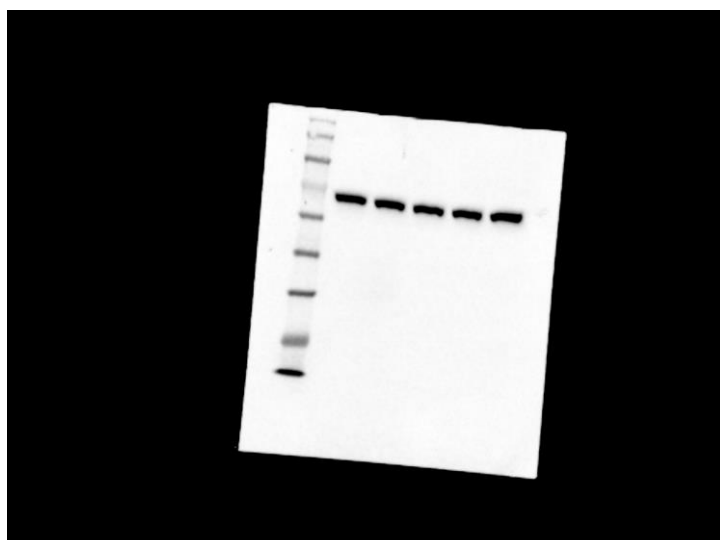

Figure5A-6

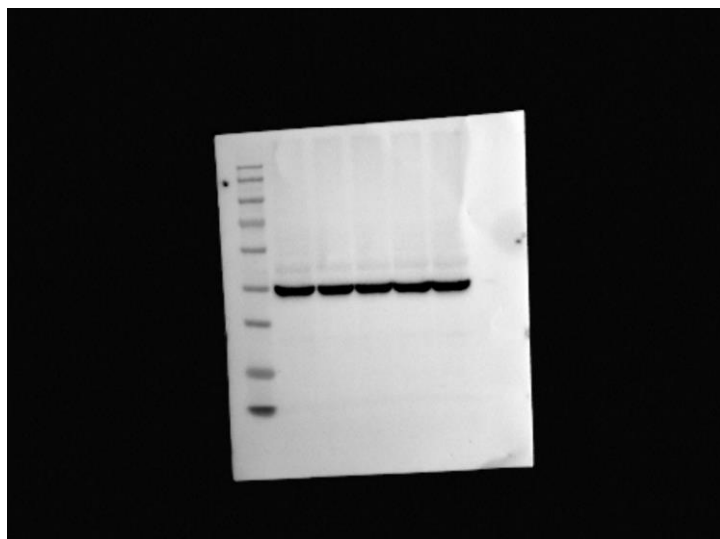

Figure5A-7

Supplement: Supplementary file 1 — Appendix S1. [file CNS-30-e14914-s001.zip › cns14914-sup-0001-AppendixS1.pdf]

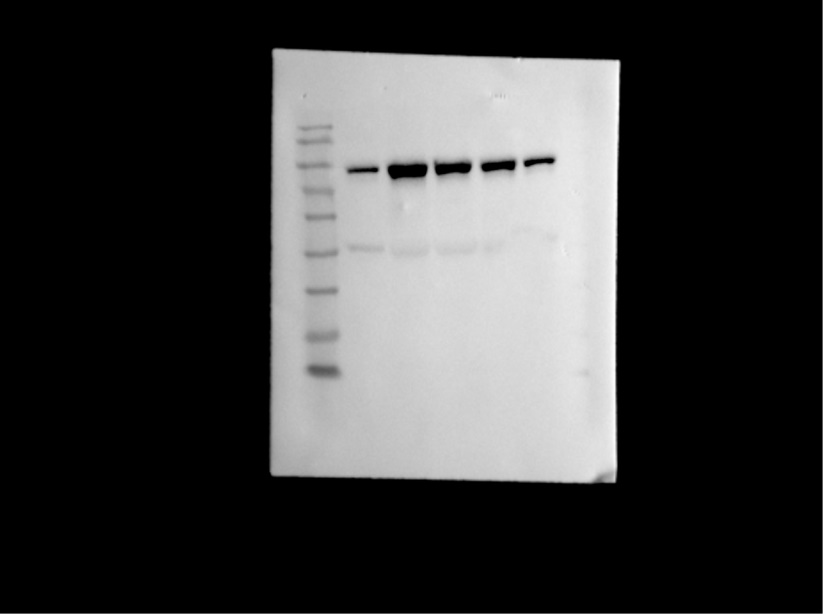


Figure4B-1


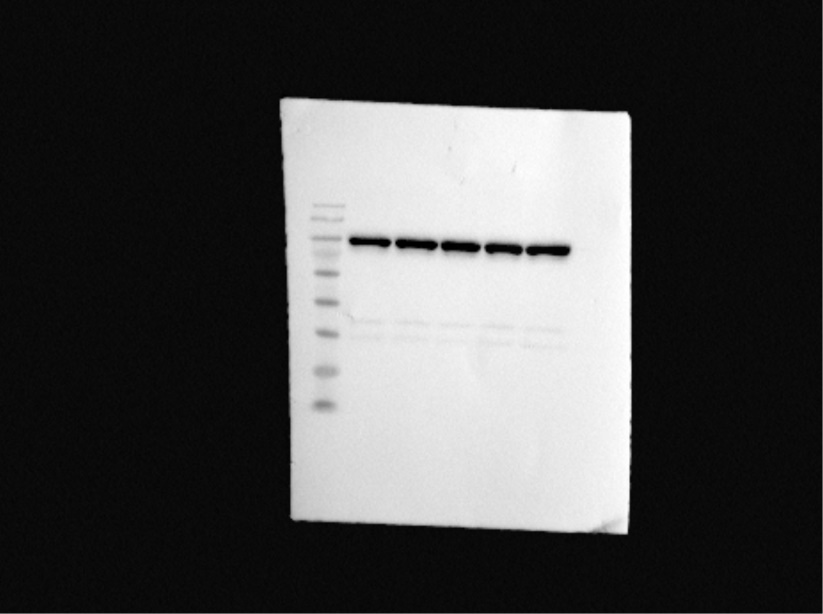


Figure4B-2


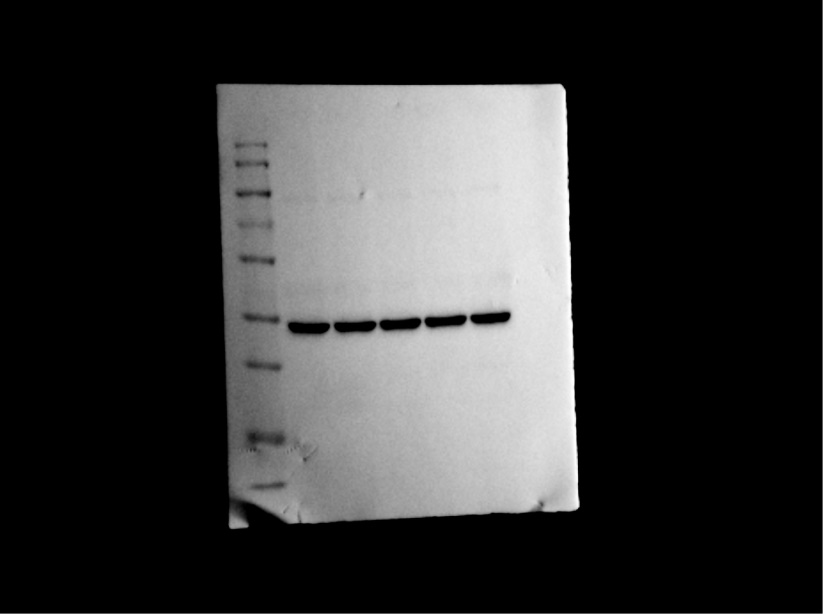


Figure4B-3


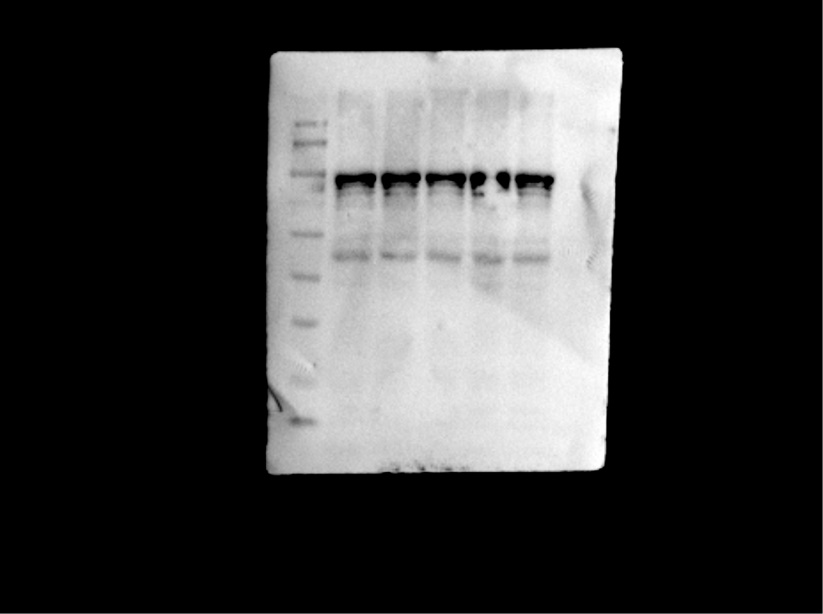


Figure5A-1


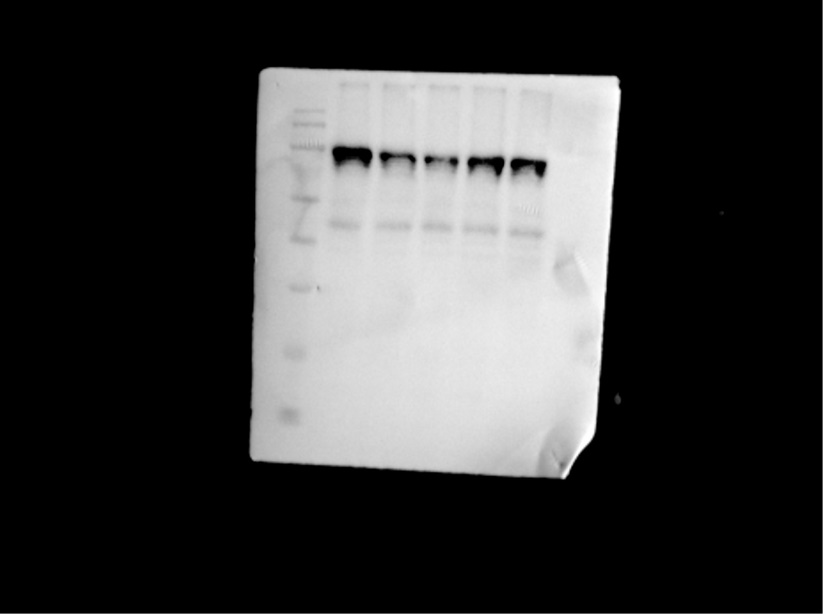


Figure5A-2


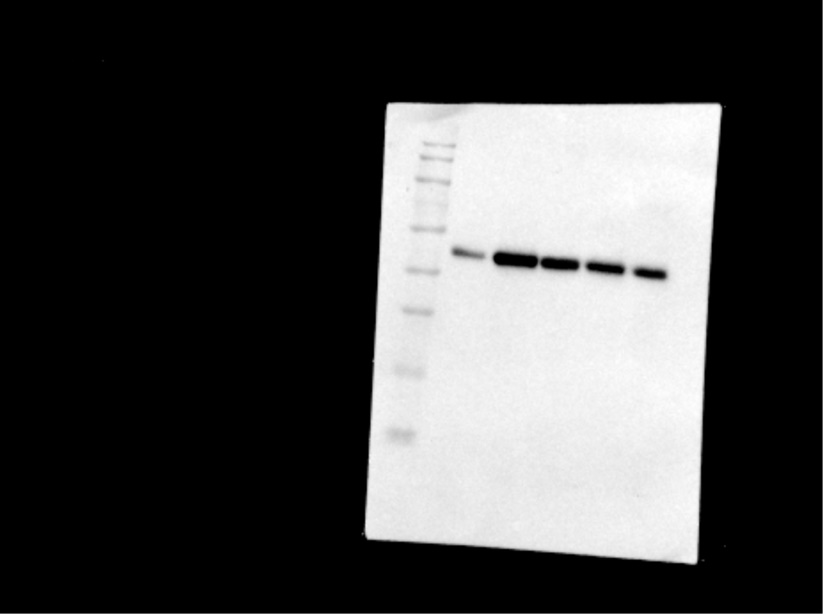


Figure5A-3


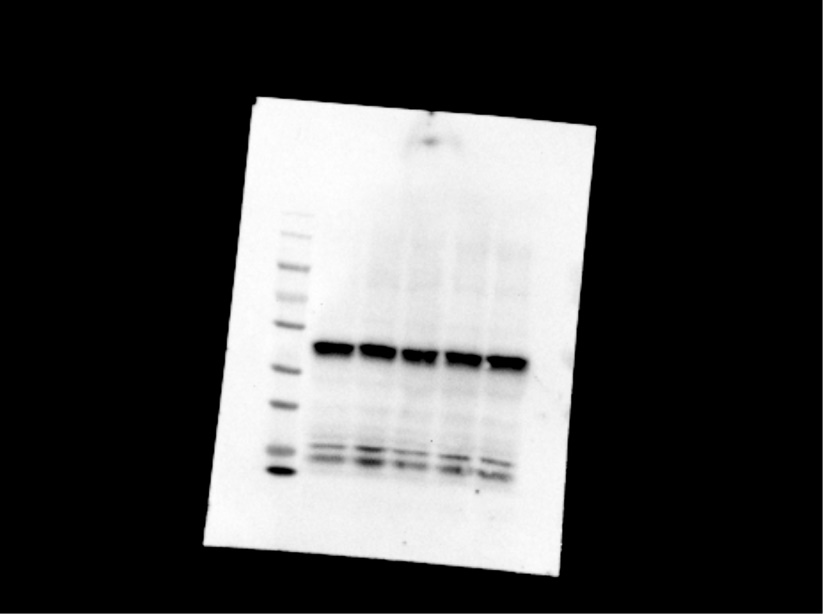


Figure5A-4


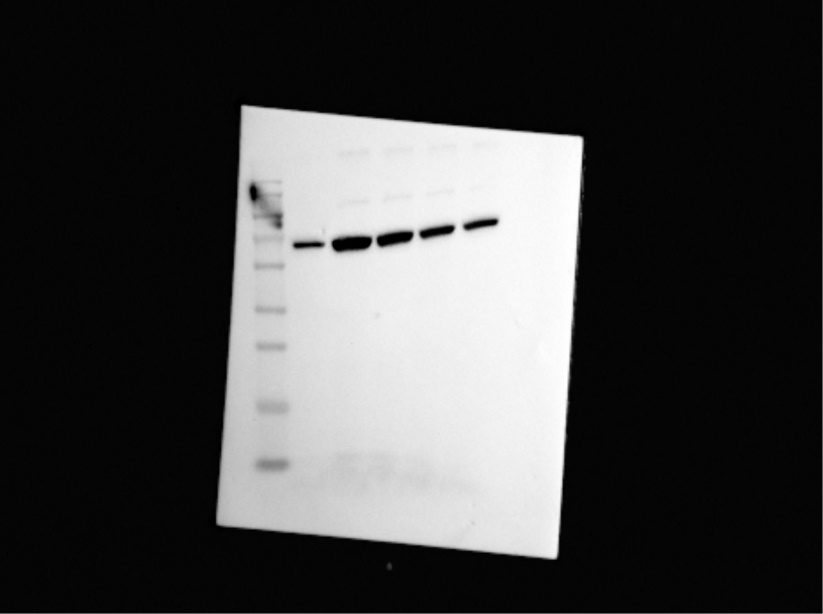


Figure5A-5


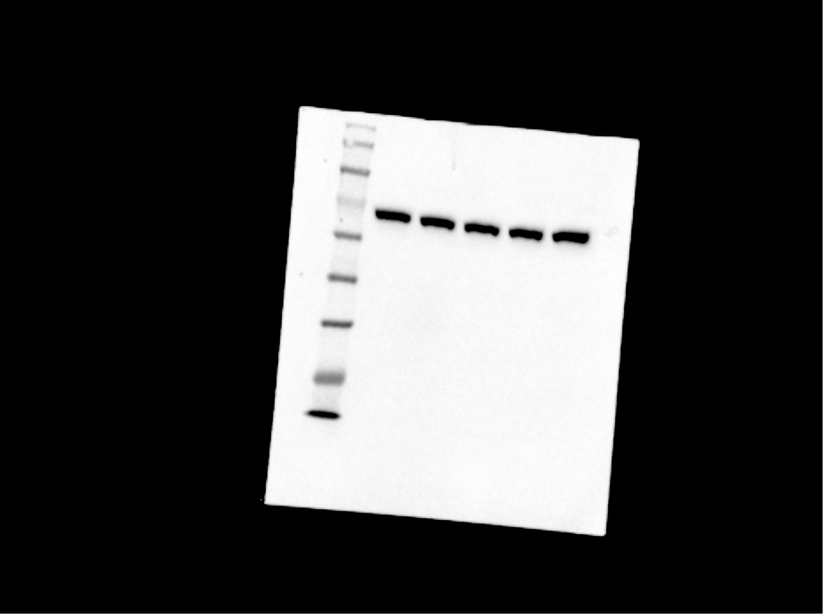


Figure5A-6


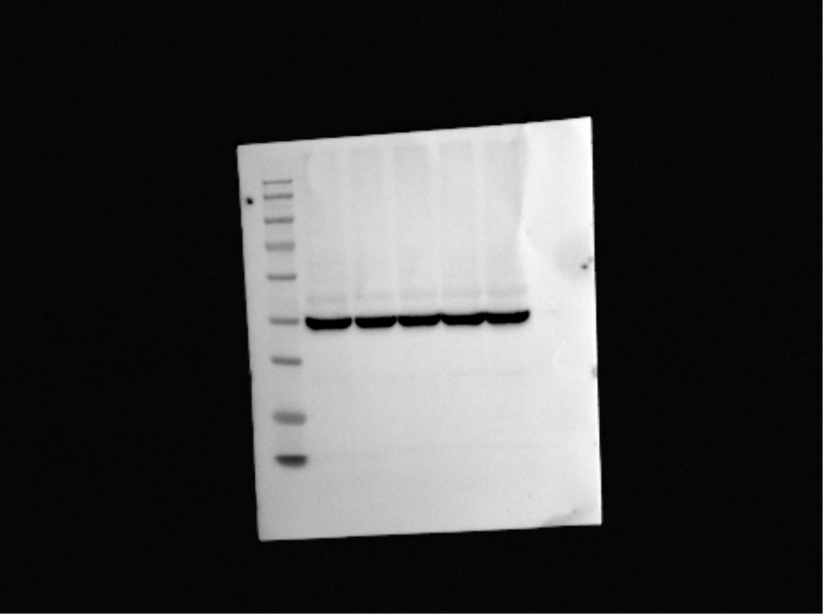


Figure5A-7

Supplement: Supplementary file 1 — Appendix S1. [file CNS-30-e14914-s001.zip › Uncropped WB Images.docx]
